# Stochastic Synchronization in Purkinje Cells with Feedforward Inhibition Could Be Studied with Equivalent Phase-Response Curves

**DOI:** 10.1186/s13408-015-0025-6

**Published:** 2015-06-19

**Authors:** Sergio Verduzco-Flores

**Affiliations:** Computational Cognitive Neuroscience Laboratory, Department of Psychology and Neuroscience, University of Colorado Boulder, Boulder, CO USA

**Keywords:** Stochastic synchrony, Cerebellum, Purkinje cells, Phase-response curve

## Abstract

Simple-spike synchrony between Purkinje cells projecting to a common neuron in the deep cerebellar nucleus is emerging as an important factor in the encoding of output information from cerebellar cortex. A phenomenon known as stochastic synchronization happens when uncoupled oscillators synchronize due to correlated inputs. Stochastic synchronization is a viable mechanism through which simple-spike synchrony could be generated, but it has received scarce attention, perhaps because the presence of feedforward inhibition in the input to Purkinje cells makes insights difficult. This paper presents a method to account for feedforward inhibition so the usual mathematical approaches to stochastic synchronization can be applied. The method consists in finding a single Phase Response Curve, called the equivalent PRC, that accounts for the effects of both excitatory inputs and delayed feedforward inhibition from molecular layer interneurons. The results suggest that a theory of stochastic synchronization for the case of feedforward inhibition may not be necessary, since this case can be approximately reduced to the case of inputs characterized by a single PRC. Moreover, feedforward inhibition could in many situations increase the level of synchrony experienced by Purkinje cells.

## Introduction

The cerebellum has a striking and relatively clear anatomical organization, which has brought hope that it could be the first brain system whose function could be understood in terms of its structure [1]. There is agreement that the cerebellum may play a role in a variety of cognitive functions, in addition to its involvement in motor control [2].

**Fig. 1:**
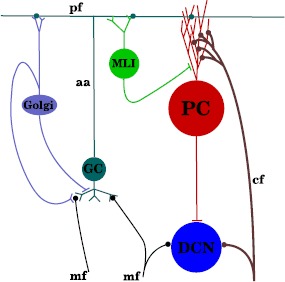
Basic connection scheme of the cerebellum. Granule cells (GC) receive afferent and efferent information from the mossy fibers (MF), and convey that information through their parallel fibers (PF). The PFs excite both Purkinje Cells (PC) and molecular layer interneurons (MLI); in turn the MLIs send axons to the PCs. Purkinje cells constitute the only output of the cerebellar cortex, and they send axons that form GABAergic inhibitory connections on the cells of the deep cerebellar nuclei (DCN). Neurons in the inferior olivary nucleus (IO) send axons known as climbing fibers (CF) which form thousands of synapses on PCs. Each PC receives excitation from a single CF. An action potential in a CF reliably causes an action potential in the PCs it innervates; these action potentials are known as complex spikes and are easily distinguishable from simple spikes, which are action potentials tonically generated by the PC, and modulated by the PFs. Not illustrated in the figure is the fact that the ascending axons (AA) of the granule cells can make multiple connections on the dendritic arbor of PCs [4], the orientation of PC dendritic arbors perpendicular to parallel fibers, or the organization in parasagittal modules [5]

Figure 1 shows the basic anatomical organization of the cerebellar cortex. See [3] or [1] for reviews. In order to designate specific types of neurons and axons in the cerebellum the abbreviations of Fig. 1 will be used. Synapses from a source neuron/axon type toward a target neuron type will be denoted by the abbreviation of the source and target connected by a dash; e.g. PF-PC denotes the synapse between parallel fibers and Purkinje cells.

Perhaps the most influential set of ideas regarding how the cerebellum works is the Marr–Albus model [6, 7], which has led to a variety of models in which Purkinje cells act like a perceptron whose learning signal comes from the climbing fibers (e.g. [8–14]). The discovery of conjunctive Long-Term Depression (LTD) in the PF-PC synapses [15–17] has added plausibility to these models. This type of LTD happens when elevated simple-spike activity occurs concurrently with climbing fiber inputs, weakening the synapses that caused the simple spikes, as hypothesized in the Albus model. Over time, however, it has become increasingly clear that conjunctive LTD alone may not explain learning in the cerebellum.

In most Marr–Albus models conjunctive LTD is the sole form of learning underlying cerebellar function. However, several studies suggest that conjunctive LTD is an incomplete explanation. First, it has been shown that cerebellar motor learning can take place in the absence of PF-PC LTD [18, 19]. Second, the correlation of Purkinje cell firing and muscle EMG can show both positive or negative correlations, with positive correlations being the more prevalent [20, 21]. If the role of Purkinje cells was to gate motor commands through just inhibition, negative correlations should be the most common. Third, synaptic inhibition of Purkinje cells, whose complex spike-elicited plasticity acts to counteract PF-PC conjunctive LTD, seems to play a role in motor learning [22], which is ignored by models that rely exclusively on PF-PC LTD. Moreover, other studies suggest that the timing of Purkinje cells’ spikes is important, not only their firing rate. Tottering mutant (tg) mice have virtually the same firing rate as that of wild types during spontaneous activity and in response to optokinetic stimulation; nevertheless, tg mutants show abnormal compensatory eye movements and severe ataxia [23].

When explaining how the timing of PC simple spikes affect their DCN targets, and how different types of CF-mediated plasticity affect cerebellar output, it may be important to pay attention to synchrony among Purkinje cells innervating the same DCN cell. This synchrony can modulate the response of the target DCN cell [24–27]. It has been observed that there exists simple-spike synchrony among PCs separated by several hundred micrometers, and that this synchrony seems to depend on afferent input [28–32]. This synchrony does not seem to be fully explained by firing rate comodulation or PC recurrent collaterals. Firing rate modulation may be insufficient in this case, because there are cases where the modulation in synchrony is unrelated to the modulation in firing rate [29, 30]. Purkinje cell recurrent collaterals tend to generate oscillations whose coherence decays with distance [33], which is inconsistent with the distances across which synchrony is found; moreover, it is unclear how sensory inputs could modify the functional coupling of Purkinje cells [31] if this coupling depended on a fast oscillatory regime. It is thus appropriate to study stochastic synchronization as a candidate mechanism to explain how Purkinje cells can activate synchronously.

The phenomenon of stochastic synchrony happens when several uncoupled oscillators synchronize their phases when receiving correlated inputs [34, 35]. The intuition behind this is that if the oscillators become entrained to the inputs then they will respond similarly, thus acquiring similar phases. An interesting aspect of stochastic synchronization is that the degree of synchrony can be controlled by the way that the oscillators respond to inputs, which opens the possibility of its modulation by plasticity mechanisms. If synchrony plays a role in shaping the response of cerebellar cortex, it seems feasible that there are plasticity mechanisms capable of creating synchrony.

One possible reason why stochastic synchrony has not been largely considered in the case of Purkinje cells is the complication arising from the feedforward inhibition in the parallel fibers. As shown in Fig. 1, PFs stimulate MLIs, which in turn stimulate PCs. This inhibition has been observed as IPSPs arising shortly after EPSPs [36], and seems to be fundamental in understanding the response of PCs [37, 38]. Although considerable advances have been made in understanding stochastic synchrony [34, 35, 39–50], no study has explored how feedforward inhibition affects this process.

Exploring stochastic synchronization of Purkinje cells requires to represent their activity in terms of their *phase*. A neuron that fires periodically can be understood as a dynamical system whose trajectory in phase space follows an asymptotically stable limit cycle. Such a dynamical system can be described by a single variable called its *phase*; the phase describes how far the current state is along the limit-cycle trajectory. Perturbations to the system (such as synaptic inputs in the case of a neuron) can be described by how they shift the phase of the system when they are received [51, 52]. The PRC (Phase Response Curve or Phase Resetting Curve) of the system plots the shift in phase that an input produces as a function of the system’s phase when the input is received. PRCs are a standard tool when understanding the behavior of coupled oscillators, and have been extensively used to describe networks of neurons [53, 54]. Also, as expected, PRCs are also a standard tool in analytical studies of stochastic synchronization.

This paper presents a prospective method to understand stochastic synchronization when feedforward inhibition is present by using an *equivalent PRC*. To model feedforward inhibition, the effect of feedforward excitation coming from the PFs to the PCs is represented with an excitatory PRC, and the effect of feedforward inhibition coming to the PCs from the MLIs is represented with an inhibitory PRC. For each excitatory input, a corresponding inhibitory input arrives after a fixed propagation delay. The equivalent PRC lumps the effect of both excitatory and delayed inhibitory PRCs so we only have one type of inputs, and yet the response of the oscillator to a spike train is similar to that when using both excitation and delayed feedforward inhibition. Studies on stochastic synchronization have not dealt with systems where there is feedforward inhibition, but the equivalent PRC allows their insights to be applied in this case.

The equivalent PRC is defined in two different ways. The first definition obtains an equivalent PRC such that the oscillator with feedforward inhibition, and the oscillator with the equivalent PRC have the same statistical distribution of their phases (this is made precise in the text). The equivalent PRC from the second definition is such that an oscillator using it and the oscillator with feedforward inhibition will have on average similar phases at particular points in time when they receive the same inputs. Monte Carlo simulations were performed to verify that oscillators using the equivalent PRCs respond similarly to an oscillator with excitatory and delayed inhibitory inputs. The results show that the oscillators with feedforward inhibition and those with equivalent PRCs produce output spike trains with a significant level of coherence, so if stochastic synchronization happens among oscillators with feedforward inhibition, it will most likely happen in oscillators with the corresponding equivalent PRCs. Furthermore, in most scenarios the effect of delayed feedforward inhibition would be a synchronizing one.

## Models

The equivalent PRCs mentioned above will be developed in the next three subsections. An equivalent PRC can only emulate the effects of excitation and delayed inhibition in an approximate manner, and there are several ways to define it. This paper presents two different definitions for the equivalent PRC. The first subsection of this section presents introductory material, and the next two subsections develop the two different definitions of the equivalent PRC.

### Reduction to a Phase Equation, and the Stationary Phase PDF

This subsection briefly outlines some basic results from [51, 52] using notation based on [39]. The results show how *N* dynamical systems oscillating in a limit cycle and receiving impulsive inputs can be represented with *N* phase variables. PRCs, the phase transition function, and the phase evolution equation are introduced for individual oscillators, which allows one to find an equation for their stationary phase Probability Density Function (PDF). If we measure the phase of the oscillator at some random point in time, the phase PDF can provide the probability that the sampled phase is in a particular interval.

Consider *N* oscillators whose dynamics can be expressed as

1 $$ \dot{\mathbf{X}}_{i}(t) = \mathbf{F}\bigl(\mathbf{X}_{i}(t) \bigr) + \mathbf{I}_{i}(t) $$

for $i= 1,\dots,N$, where the vector $\mathbf{X}_{i}(t)$ denotes the state of oscillator *i* at time *t*, **F** is the function describing the dynamics of each oscillator, and $\mathbf{I}_{i}(t)$ represents external random inputs consisting of impulsive displacements in phase space. We assume that the oscillators have an asymptotically stable limit cycle $\mathbf{X}_{0}(t)$. The impulsive inputs are given by

2 $$ \mathbf{I}_{i}(t) = \sum_{n=1}^{\infty}\mathbf{e}_{n}^{i} \delta\bigl(t - t_{n}^{i} \bigr), $$

where $t_{n}^{i}$ represents the time of the *n*th input to the *i*th oscillator, and $\mathbf{e}_{n}^{i}$ provides the direction of the shift in phase space caused by the corresponding input, so that at time $t_{n}^{i}$ the oscillator *i* receives an immediate shift in phase space from point $\mathbf{X}_{i}$ to point $\mathbf{X}_{i} + \mathbf{e}_{n}^{i}$. Since our oscillator represents a neuron, the value of $\mathbf{e}_{n}^{i}$ should be determined by the synapse that receives the input. Furthermore, it is assumed that the inputs will not take the system outside the basin of attraction of $\mathbf{X}_{0}(t)$. The inputs that will be considered in this paper behave as Poisson random point processes. If an input has a mean firing rate *r*, then its interimpulse interval *T* has an exponential distribution:

3 $$ P(T) = r \mathrm{e}^{-rT}, $$

where $P(T)$ is the probability density function for *T*. Although Poisson point processes constitute a reasonable null hypothesis regarding the statistical structure of inputs to Purkinje cells, this hypothesis may not always be valid [55].

We define a phase variable *θ* along the limit cycle so that $\theta(t) = \theta(\mathbf{X}_{0}(t))$, *θ* has a constant angular velocity *ω*, and its range is $[0,1)$. This means that in the absence of external inputs we will have

4 $$ \dot{\theta}_{i}(t) = \omega_{i}. $$

In the first part of the results section we work with systems where all the oscillators have the same angular frequency *ω*. The phase of points not directly on $\mathbf{X}_{0}$ is defined through the use of isochrons, which are the set of points that asymptotically converge to a particular trajectory $\mathbf{X}^{*}_{0}$ in the periodic orbit. If the points on an isochron converge to the trajectory $\mathbf{X}^{*}_{0}$, then their phase at time *t* is $\theta(\mathbf{X}^{*}_{0}(t))$. For all functions in this paper whose arguments include a phase value, I assume that this phase value is taken modulo 1; e.g. phases 0, 1 are the same phase, and the same can be said of phases −0.1, 0.9, and 2.9. When an oscillator has phase *θ* at time *t*, and an input shifts its state at that moment by an amount **e** then this state moves to a new isochron, with the consequent shift in phase denoted by $G(\theta,\mathbf{e})$. We define the phase transition function as

5 $$ F(\theta,\mathbf{e}) = \theta + G(\theta,\mathbf{e}), $$

with its output taken modulo 1. If the phase of an oscillator at time $t_{n}$ right before its *n*th input is $\theta_{n}$, then the phase right after the input can be written as $\phi_{n} \equiv F(\theta_{n},\mathbf{e}_{n}) = \theta_{n} + G(\theta_{n},\mathbf{e}_{n})$. The evolution equation of the phase is an iterative equation describing the phase of the oscillator at the time when the $(n+1)$th input arrives:

6 $$ \theta_{n+1} = \omega T_{n} + F(\theta_{n}, \mathbf{e}_{n}) = \theta_{n} + \omega T_{n} + G( \theta_{n},\mathbf{e}_{n}), $$

where $T_{n} = t_{n+1}-t_{n}$. The dynamics of the phase in continuous time are described by the equation

7 $$ \dot{\theta}(t) = \omega + \sum_{n=1}^{\infty}G(\theta_{n},\mathbf{e}_{n}) \delta(t-t_{n}). $$

Given that the input times $t_{n}$ and the input effects $\mathbf{e}_{n}$ are random variables, so are the phases $\theta_{n}$. The Probability Density Function (PDF) of the phase *θ* at time step *n* is denoted $\rho(\theta,n)$. This PDF can be described by the following generalized Frobenius–Perron equation [56]:

8 $$ \rho(\theta,n+1) = \int_{0}^{1} W(\theta - \phi) \int Q(\mathbf{e}) \int_{0}^{1} \delta\bigl(\phi - F(\psi,\mathbf{e})\bigr)\rho(\psi,n)\,d\psi \,d\mathbf{e}\,d\phi. $$

The term $W(\theta - \phi)$ is a density function for the input interimpulse interval $T_{n}$ expressed as a periodized change of phase with magnitude $\theta-\phi$ (see Eq. (9)). $Q(\mathbf{e})$ is the probability density function for **e**. Intuitively, the two innermost integrals produce the expected phase after the *n*th input with *ψ* being the starting phase; this expected phase, represented by the integration variable *ϕ* is taken in the outermost integral in order to calculate the probability that during the interspike interval the phase transitions from *ϕ* to *θ*. The transition kernel $W(\theta)$ can be explicitly obtained in the case of Poisson inputs, considering that its arguments are taken to be modulo 1:

9 $$\begin{aligned} W(\theta) &= \frac{1}{\omega} \sum_{j=0}^{\infty}P \biggl( \frac{\theta+j}{\omega} \biggr) = \frac{r}{\omega} \sum _{j=0}^{\infty}\mathrm{e}^{-({r}/{\omega})\theta} \mathrm{e}^{-({r}/{\omega})j} \\ &= \frac{A \mathrm{e}^{-A \theta}}{1 - \mathrm{e}^{-A}}, \end{aligned}$$

with $A = r/\omega$. In the limit of a large number of transitions the PDFs $\rho(\theta,n)$ will reach a stationary state $\rho(\theta)$ obeying:

10 $$ \rho(\theta) = \int_{0}^{1} W(\theta - \phi) \int Q(\mathbf{e}) \int_{0}^{1} \delta\bigl(\phi - F( \psi,\mathbf{e})\bigr)\rho(\psi)\,d\psi \,d\mathbf{e}\,d\phi. $$

I refer to this equation as the phase PDF equation.

### The Equivalent PRC as a Function of the Phase PDF

The first idea to obtain an equivalent PRC, denoted in this subsection as *Δ*, is to take the phase PDF $\rho(\theta)$ produced by the excitatory and inhibitory inputs, and define *Δ* so its phase PDF matches $\rho(\theta)$. In practice, the formulas developed in this section allow one to take a numerically or experimentally measured phase PDF $\rho(\theta)$, and obtain a single PRC *Δ* so that with a single Poisson input, *Δ* produces a phase PDF similar to $\rho(\theta)$. The formulas from this section are tested by numerically obtaining the phase PDF created by an oscillator with feedforward inhibition, and comparing it with the phase PDF created by an oscillator using the equivalent PRC.

I start by assuming a single oscillator and a single Poisson process that produces excitatory inputs. Feedforward inhibition is modeled by assuming that for each excitatory input at time $t_{n}$ there will be a corresponding inhibitory input at time $t_{n} + d$, where *d* represents the feedforward delay. All excitatory inputs will produce a shift in phase space $\mathbf{e}_{\mathrm{exc}}$, whereas inhibitory inputs produce a shift $\mathbf{e}_{\mathrm{inh}}$. The excitatory and inhibitory PRCs are defined, respectively, as $\varDelta _{\mathrm{exc}}(\theta) = G(\theta,\mathbf{e}_{\mathrm{exc}})$, $\varDelta _{\mathrm{inh}}(\theta) = G(\theta,\mathbf{e}_{\mathrm{inh}})$, where the function *G* maps shifts in phase space to shifts in phase. An oscillator using instead the equivalent PRC will present a shift in phase $\varDelta (\theta)$ at the times when the excitatory inputs arrive.

I assume a perturbation from the system where the PRC is zero for all phases and the phase PDF is uniform. Furthermore, the perturbation is small enough so that the phase transition function *F* is still invertible. Let $\rho(\theta) = 1 + \varepsilon \rho_{1}(\theta)$, where *ε* is a scalar that determines the size of the perturbation. Expand the equivalent PRC as $\varDelta (\theta) = \varepsilon \varDelta _{1}(\theta) + \varepsilon^{2} \varDelta _{2}(\theta) + O(\varepsilon^{3})$ (using big Oh notation), so that as *ε* goes to zero we recover the unperturbed system. Before substituting these terms, the phase PDF equation (10) is simplified in three steps:

1. The middle integral disappears. There is only one type of input, so the PDF *Q* is a delta function that can be integrated out.
2. We perform the innermost integral using the basic formula for performing change of variables with Dirac *δ* functions. If $g(x)$ is a real function with a root at $x_{0}$ the formula is $\delta(g(x)) = \frac{\delta(x - x_{0})}{\vert g'(x_{0})\vert }$.
3. We substitute the transition kernel *W* for its expression in Eq. (9).

This yields

11 $$ \rho(\theta) = \int_{0}^{1} \frac{A \mathrm{e}^{-A(\theta-\phi)}}{1 - \mathrm{e}^{-A}} \frac{\rho(F^{-1}(\phi))}{\vert F'(F^{-1}(\phi))\vert }\,d\phi. $$

Notice that the argument $(\theta - \phi)$ of *W* is still taken modulo 1, so we need separate integrals for the cases when this argument is positive and negative:

12 $$ \begin{aligned}[b] \rho(\theta) = {}&\frac{A \mathrm{e}^{-A \theta}}{1 - \mathrm{e}^{-A}} \biggl[ \int_{0}^{\theta} \mathrm{e}^{A \phi} \frac{\rho(F^{-1}(\phi))}{\vert F'(F^{-1}(\phi))\vert }\,d\phi\\ &{} + \mathrm{e}^{-A} \int _{\theta}^{1} \mathrm{e}^{A \phi} \frac{\rho(F^{-1}(\phi))}{\vert F'(F^{-1}(\phi))\vert } \,d\phi \biggr]. \end{aligned} $$

We now assume $F(\phi) = \phi + \varepsilon \varDelta _{1}(\phi) + \varepsilon^{2} \varDelta _{2}(\phi) + O(\varepsilon^{3})$. Let $F^{-1}(\phi) = \phi + \varepsilon \psi_{1}(\phi) + \varepsilon^{2} \psi_{2}(\phi) + O(\varepsilon^{3})$. Substituting these two previous expressions in the identity $F(F^{-1}(\phi)) = \phi$ we obtain

$$\begin{aligned} \psi_{1} &= -\varDelta _{1}, \\ \psi_{2} &= -\varDelta _{2} + \varDelta _{1} \varDelta _{1}', \\ F^{-1}(\phi) &= \phi - \varepsilon \varDelta _{1} + \varepsilon^{2} \bigl(\varDelta _{1} \varDelta _{1}' - \varDelta _{2}\bigr) + O\bigl(\varepsilon^{3}\bigr), \end{aligned}$$

where the argument has been omitted in some functions for brevity of notation. Using this expression for $F^{-1}$ we find

$$ \frac{\rho(F^{-1}(\phi))}{\vert F'(F^{-1}(\phi))\vert } = \frac{1 + \varepsilon \rho_{1} - \varepsilon^{2} \varDelta _{1} \rho_{1}' + O(\varepsilon^{3})}{ 1 + \varepsilon \varDelta _{1}' - \varepsilon^{2}(\varDelta _{2}' - \varDelta _{1} \varDelta _{1}'') + O(\varepsilon^{3})}, $$

where we use the fact that since *F* is invertible, $F'>0$. Using long division to eliminate the quotient and substituting into (12) yields

13 $$ \begin{aligned}[b] 1 + \varepsilon \rho_{1}(\theta) = {}&\frac{A \mathrm{e}^{-A \theta}}{1 - \mathrm{e}^{-A}} \biggl( \int_{0}^{\theta} \mathrm{e}^{A \phi} \bigl[ 1 + \varepsilon\bigl(\rho_{1} - \varDelta _{1}' \bigr) \\ &{}- \varepsilon^{2} \bigl(\varDelta _{1} \rho_{1}' - \varDelta _{1} \varDelta _{1}'' + \varDelta _{1}' \rho_{1} - \bigl( \varDelta _{1}'\bigr)^{2} + \varDelta _{2}' \bigr) \bigr]\,d\phi \\ &{}+\mathrm{e}^{-A} \int_{\theta}^{1} \mathrm{e}^{A \phi} \bigl[ 1 + \varepsilon\bigl(\rho_{1} - \varDelta _{1}'\bigr) \\ &{}- \varepsilon^{2} \bigl( \varDelta _{1} \rho_{1}' - \varDelta _{1} \varDelta _{1}'' + \varDelta _{1}' \rho_{1} - \bigl(\varDelta _{1}' \bigr)^{2} + \varDelta _{2}'\bigr) \bigr]\,d\phi \biggr). \end{aligned} $$

The terms in this equation can be grouped according to the power of *ε* that they contain. The zeroth-order terms yield an identity corresponding to the unperturbed case. The equation corresponding to the first power of *ε* is

14 $$ \rho_{1}(\theta) = \frac{A \mathrm{e}^{-A \theta}}{1 - \mathrm{e}^{-A}} \biggl( \int_{0}^{\theta} \mathrm{e}^{A \phi} \bigl(\rho_{1} - \varDelta _{1}' \bigr)\,d\phi + \mathrm{e}^{-A} \int_{\theta}^{1} \mathrm{e}^{A \phi} \bigl(\rho_{1} - \varDelta _{1}' \bigr)\,d\phi \biggr). $$

This equation can be differentiated with respect to *θ*, and in the resulting equation we can solve for $\varDelta _{1}'$ to obtain

$$ \varDelta _{1}'(\theta) = -\frac{1}{A} \rho_{1}'(\theta). $$

Integrating this provides an expression to evaluate $\varDelta _{1}$:

15 $$ \varDelta _{1}(\theta) = \frac{1}{A} \bigl(C_{1} - \rho_{1}(\theta)\bigr), $$

where $C_{1}$ is an integration constant. Notice that equation (14) only provides constraints for the derivative of $\varDelta _{1}$, so it cannot be used to determine the value of $C_{1}$, reflecting the fact that oscillators with different frequencies can have the same stationary phase PDF. Since the quantity $\varepsilon C_{1}/A$ becomes a constant term in *Δ*, it adds an extra amount of advance or retardation to the phase whenever an input is received, and we can adjust its value so that the mean firing rate of the oscillator with two PRCs matches that of the oscillator with the equivalent PRC.

The equation corresponding to $\varepsilon^{2}$ in Eq. (13) is

16 $$ \begin{aligned}[b] &\int_{0}^{\theta} \mathrm{e}^{A \phi} \bigl[ \varDelta _{1} \bigl(\rho_{1}' - \varDelta _{1}''\bigr) + \varDelta _{1}' \bigl(\rho_{1} - \varDelta _{1}'\bigr) + \varDelta _{2}'\bigr]\,d\phi \\ &\quad = - \mathrm{e}^{-A} \int _{\theta}^{1} \mathrm{e}^{A \phi} \bigl[ \varDelta _{1} \bigl(\rho_{1}' - \varDelta _{1}''\bigr) + \varDelta _{1}' \bigl(\rho_{1} - \varDelta _{1}'\bigr) + \varDelta _{2}'\bigr]\,d\phi. \end{aligned} $$

As in the previous case we can differentiate with respect to *θ* and solve for $\varDelta _{2}'$, which gives

17 $$ \varDelta _{2}' = \frac{1}{A} \biggl[ \rho_{1} \rho_{1}' - C_{1} \rho_{1}' - \frac{1}{A}\bigl(C_{1} \rho_{1}'' + \rho_{1} \rho_{1}''\bigr) + \rho_{1} \rho_{1}' + \frac{1}{A}\bigl(\rho_{1}' \bigr)^{2} \biggr]. $$

We can use the integration by parts formula $\int \rho_{1} \rho_{1}'' = \rho_{1} \rho_{1}' - \int (\rho_{1}')^{2}$ to find the antiderivative of this expression, which is

18 $$ \varDelta _{2} = \frac{1}{A} \biggl[ \rho_{1} \biggl( \rho_{1} + \frac{\rho_{1}'}{A} - C_{1} \biggr) - \frac{C_{1}}{A} \rho_{1}' + C_{2} \biggr], $$

where $C_{2}$ is an integration constant. Alternatively, we can avoid having another integration constant by finding the definite integral of Eq. (17) from 0 to *θ*, obtaining

19 $$ \begin{aligned}[b] \varDelta _{2} = {}&\frac{1}{A} \biggl[ \rho_{1} \biggl( \rho_{1} + \frac{\rho_{1}'}{A} - C_{1} \biggr) - \rho_{1}(0) \biggl(\rho_{1}(0) + \frac{\rho_{1}'(0)}{A} - C_{1} \biggr) \\ &{}- \frac{C_{1}}{A} \bigl(\rho_{1}' - \rho_{1}'(0)\bigr) \biggr]. \end{aligned} $$

Equations (15) and (19) provide the relationship between a phase PDF, and the PRC that lead to it. Notice that these results are consistent with those of [43], where a perturbative expansion is used to go from the PRC to the phase PDF, instead of going from the phase PDF to the PRC, as done here.

Monte Carlo simulations were performed in order to verify that an oscillator using the *Δ* approximation from the formulas above would respond similarly to an oscillator using $\varDelta _{\mathrm{exc}}$ and $\varDelta _{\mathrm{inh}}$ when provided with the same input, which consisted of a Poisson spike train with a frequency $r = 600\mbox{ Hz}$. This high rate comes from the assumption that the input comes from many homogeneous synapses receiving spike trains at a lower rate. Notice that the superposition of Poisson spike trains is also a Poisson spike train, whose rate is the sum of the rates for the superposed trains. The case of heterogeneous synapses will be treated further ahead. It is assumed that when the oscillator’s phase transitions from 1 to 0 a spike is emitted; phase is not allowed to go from 0 to 1 due to inhibition. The shape of $\varDelta _{\mathrm{exc}}$ was generated as a scaled version of the function $1 - \cos(2\pi x)$, and $\varDelta _{\mathrm{inh}}$ is a scaled version of $\cos(2\pi x) - 1$, corresponding to type I PRCs [57]. The general shape of $\varDelta _{\mathrm{exc}}$ is justified by the measurements that have been taken of Purkinje cells’ PRCs at high frequencies [58], indicating that this is a positive, unimodal, type I PRC. The shape of $\varDelta _{\mathrm{inh}}$ is justified by noticing that stimulation of MLIs tends to consistently induce an increase in the latency of the next spike in Purkinje cells [36, 59]. Although the specific shapes of $\varDelta _{\mathrm{exc}}$ and $\varDelta _{\mathrm{inh}}$ used here may be somewhat different from that in real cells, the formulas in this paper can nevertheless be applied when the excitatory and inhibitory PRCs have any smooth shape. The phase PDF required in Eqs. (15) and (19) were obtained by sampling the phase whenever an input arrived. The phase histogram corresponding to these samples was normalized and smoothed with the Savitzky–Golay method. The derivative of the phase PDF was obtained using a 2 point rule with the smoothed phase PDF histogram. The constant $C_{1}$ of Eq. (15) was adjusted for each simulation up to two decimal places so that the firing rates of the oscillators with one and two PRCs would match for an input rate of 600 Hz.

The derivation of the formulas for *Δ* requires that the amplitudes $a_{\mathrm{exc}}$ of $\varDelta _{\mathrm{exc}}$ and $a_{\mathrm{inh}}$ of $\varDelta _{\mathrm{inh}}$ be moderate. The performance of the formulas gradually deteriorates with larger amplitudes. The values of $a_{\mathrm{exc}}$ and $a_{\mathrm{inh}}$ were chosen to be near the limit where the agreement obtained from using *Δ* is still acceptable. The feedforward delay *d* is taken to be 5 ms, which is somewhat larger than usual estimations of 1 or 2 ms. This long delay is used to test the limitations of the approaches used here to obtain an equivalent PRC, particularly the ones to be presented in the next subsection. Considering that in the absence of inputs the oscillators have a frequency of 50 Hz, a delay of 5 ms constitutes one quarter of the period, which is a considerable interval.

**Fig. 2:**
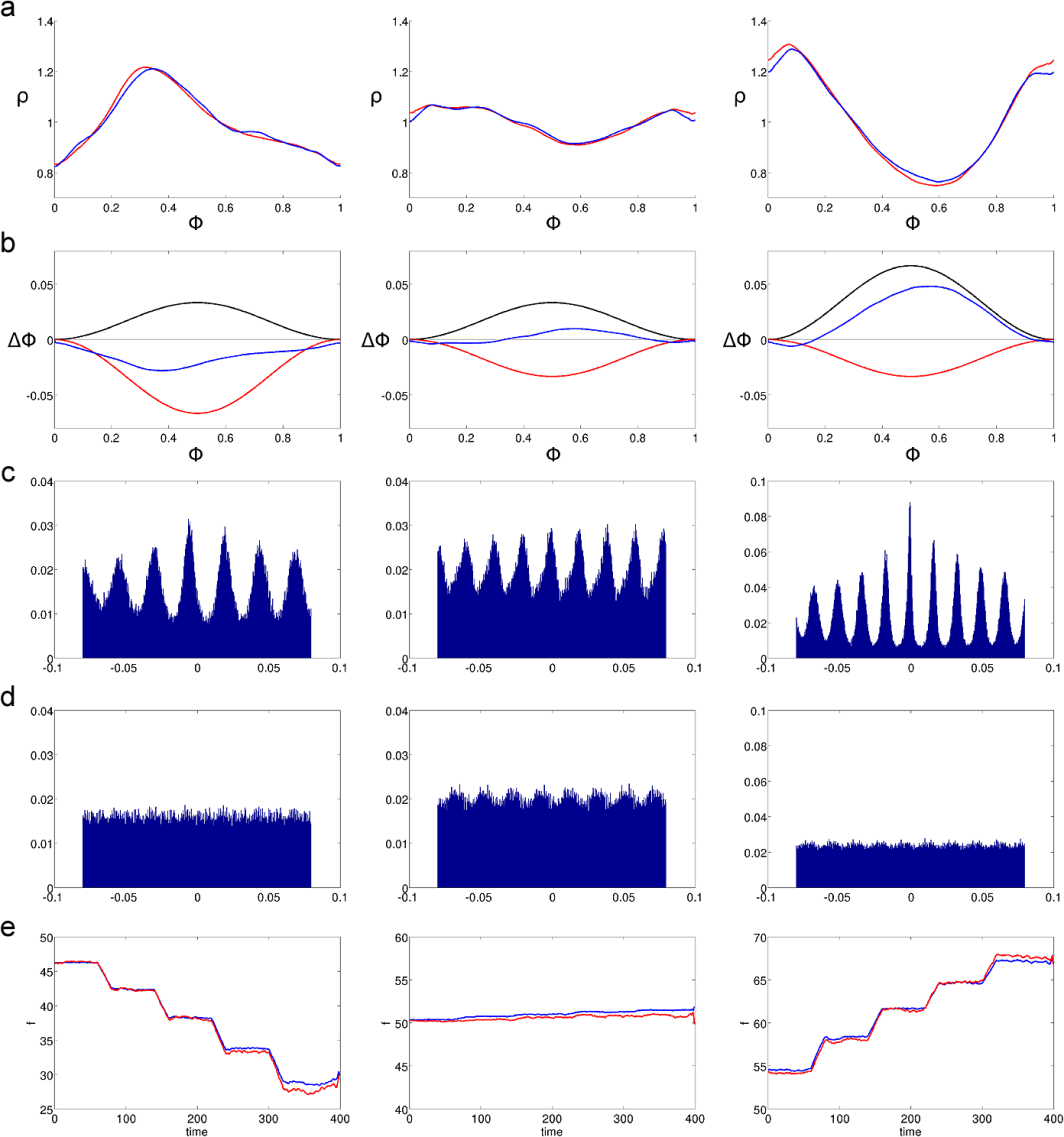
Comparison of oscillators with two and one PRCs when matching phase PDFs. Simulations for three different amplitude combinations of $\varDelta _{\mathrm{exc}}$ and $\varDelta _{\mathrm{inh}}$. For all *rows*, *the figures on the left* correspond to $a_{\mathrm{exc}} = 1/30$, $a_{\mathrm{inh}} = 2/30$, *figures on the middle* correspond to $a_{\mathrm{exc}} = 2/30$, $a_{\mathrm{inh}} = 2/30$, and *figures on the right* correspond to $a_{\mathrm{exc}} = 2/30$, $a_{\mathrm{inh}} = 1/30$. The input rate for all simulations in **a**–**d** was $r = 600\mbox{ Hz}$. **a**: Stationary phase PDFs for oscillators with feedforward inhibition (*red*) and with their equivalent PRC (*blue*). **b**: PRCs used in the simulation. $\varDelta _{\mathrm{exc}}$ (*black*), $\varDelta _{\mathrm{inh}}$ (*red*), *Δ* (*blue*). **c**: Cross-correlograms of the oscillators’ output spike trains with one and two PRCs. *The vertical axis* corresponds to normalized spike count per time bin, and *the horizontal axis* to time shift. Normalization was done by dividing the bin spike counts between the total number of spikes in the output of the oscillator with two PRCs. **d**: Cross-correlograms of the output spike train of the oscillator with two PRCs and a periodic spike train with the same mean frequency. Normalization was done as in **c**. **e**: Firing rate response of the oscillators with two (*red*) and one (*blue*) PRCs to five different levels of input rates. Input rates range from 240 Hz to 1200 Hz in constant increments of 240 Hz

Figure 2 presents the results of substituting the two PRCs $\varDelta _{\mathrm{exc}}$ and $\varDelta _{\mathrm{inh}}$ by the equivalent PRC *Δ*. Simulations were performed for three cases, each corresponding to a column in Fig. 2. The first case is when $a_{\mathrm{exc}} < a_{\mathrm{inh}}$, the second case when $a_{\mathrm{exc}} = a_{\mathrm{inh}}$, and the third case is for $a_{\mathrm{exc}} > a_{\mathrm{inh}}$. Each of these three cases produces a different shape for the equivalent PRC (panel b). Panel a shows that there is a good match between the stationary phase PDFs, as would be expected since the formulas for *Δ* were derived with this result in mind. The PDF curves come from 400 seconds of simulation, which permitted on average 240000 sample points.

It seems reasonable that the output spikes of the oscillators with one and two PRCs would come at similar times, since they have similar rates and phase PDFs. This is what is shown in panel c of Fig. 2, which shows the cross-correlograms of the output spike trains for both oscillators. The height of each bin in the cross-correlograms can be interpreted as the fraction of the spikes in the oscillator with two PRCs that coincide with a spike of the oscillator with one PRC. For comparison purposes similar cross-correlograms were produced in panel d, between the spike trains of the oscillators with two PRCs and a spike train with the same frequency and constant interspike intervals. It should be remembered that the parameter $C_{1}$ was adjusted so that both oscillators would have the same frequency, but from panel d it is apparent that the peaks in the top cross-correlograms are not just a product of periodicity in the signals.

Finally, I compared the response of both oscillators when the input firing rates were changed through the simulation. As can be seen in panel e, slight inaccuracies in the calculation of the $C_{1}$ parameter were amplified for larger firing rates. Moreover, in the case of balanced excitatory and inhibitory amplitudes the output firing rate tended to increase for larger input firing rates. This effect is amplified for larger PRC amplitudes, reflecting the fact that if the phase shift of $\varDelta _{\mathrm{exc}}$ is large, the oscillator will spike before the inhibition arrives.

So far it has been assumed that all inputs of the same type (excitatory or inhibitory) will produce the same effect on the oscillator. On the other hand, a real neuron tends to have heterogeneous synapses. One way to represent this is to have separate excitatory and inhibitory PRCs for each input, representing different synapses. If an oscillator has $N_{\mathrm{syn}}$ different inputs, we will have PRCs $\varDelta _{\mathrm{exc}}^{i}$, $\varDelta _{\mathrm{inh}}^{i}$, for $i = 1,\dots,N_{\mathrm{syn}}$. In this case, for each excitatory/inhibitory pair we may create an equivalent PRC $\varDelta ^{i}$. One approach to create $\varDelta ^{i}$ is to consider the stationary phase PDF $\rho^{i}$ and output firing rate that would be produced if the inputs with $\varDelta _{\mathrm{exc}}^{i}$, $\varDelta _{\mathrm{inh}}^{i}$ were considered in isolation. The formulas above could then be used to create $\varDelta ^{i}$. Instead of doing this, in the next subsection I develop a different way of obtaining $\varDelta ^{i}$, based on a more direct calculation of the phase-shifting effects produced by feedforward inhibition.

### Obtaining an Equivalent PRC Using the Expected Inhibition

For the purposes of this paper, the ideal result of an equivalent PRC would be to have identical output spike trains for the oscillator with feedforward inhibition, and for the oscillator using the equivalent PRC. This would cause all the effects of stochastic synchronization to be the same for both oscillators. The method presented above to obtain *Δ* is based on creating an oscillator with a single PRC that has the same phase PDF as the one with two PRCs. However, having the same phase PDF and firing rate as the oscillator with feedforward inhibition does not guarantee that the output spike trains of the two oscillators will be the same. Indeed, given a phase PDF and a firing rate, there are many spike trains satisfying these two conditions. The equivalent PRC *Δ* defined above is therefore not necessarily optimal, and this raises the question on whether we can find an alternative definition for an equivalent PRC.

To obtain an equivalent PRC we could take a more direct approach, again trying to ensure that the phases of the oscillators with one and two PRCs are often the same. To develop this idea, let us consider two oscillators $O_{\mathrm{ei}}$ and $O_{\mathrm{eq}}$. $O_{\mathrm{ei}}$ experiences delayed feedforward inhibition, with excitatory and inhibitory PRCs $\varDelta _{\mathrm{exc}}$ and $\varDelta _{\mathrm{inh}}$. On the other hand $O_{\mathrm{eq}}$ has only an equivalent PRC $\varDelta _{\mathrm{eq}}$, and receives its inputs at the times when $O_{\mathrm{ei}}$ receives excitatory spikes. At time *t* we can denote the phase of $O_{\mathrm{ei}}$ by $\phi(t)$, and the phase of $O_{\mathrm{eq}}$ by $\phi_{\mathrm{eq}}(t)$. Let us assume that at time $t_{0}$ an excitatory input is received by $O_{\mathrm{ei}}$, and the corresponding inhibitory input is received at time $t_{0} + d$. The phase of $O_{\mathrm{ei}}$ right before the inhibitory input at time $t_{0}+d$ can be denoted as $\phi(t_{0}+d)$, and the phase right after the inhibitory input as $\phi^{+}(t_{0}+d)$, so that $\phi^{+}(t_{0}+d) = \phi(t_{0}+d) + \varDelta _{\mathrm{inh}}(\phi(t_{0}+d))$. We could define $\varDelta _{\mathrm{eq}}$ with the aim that the input at time $t_{0}$ creates a phase shift in $\phi_{\mathrm{eq}}$ such that $\phi_{\mathrm{eq}}(t_{0}+d) = \phi^{+}(t_{0}+d)$.

A brief consideration of this goal shows that it is impossible to achieve in an exact manner when the inputs are random. In the period between $t_{0}$ and $t_{0}+d$ there may be several inputs shifting the phase *ϕ*, and the magnitude of the inhibitory shift $\varDelta _{\mathrm{inh}}(\phi(t_{0}+d))$ depends on what the phase is in that moment. In other words, $\varDelta _{\mathrm{inh}}(\phi(t_{0}+d))$ is a random variable, and $\varDelta _{\mathrm{eq}}$ must shift the phase $\phi_{\mathrm{eq}}(t_{0})$ without knowing its value. Another important consideration about this goal is that the equivalent PRC that we are trying to obtain will generate a phase PDF that should be somewhat different from the one arising in the oscillator with two PRCs. This comes from the fact that at time $t_{0}$ the phase shifts from $\varDelta _{\mathrm{eq}}$ and $\varDelta _{\mathrm{exc}}$ will be different, so the time spent in the phases near $\phi(t_{0})$ should be different.

The approach taken in this subsection to obtain $\varDelta _{\mathrm{eq}}$ is based on the following idea:

20 $$ \varDelta _{\mathrm{eq}}(\phi) \equiv \varDelta _{\mathrm{exc}}(\phi) + E \bigl[ \varDelta _{\mathrm{inh}}\bigl(\phi(t_{0}+d)\bigr) \bigr], $$

where $E [\varDelta _{\mathrm{inh}}(\phi(t_{0}+d)) ]$ is the expected value of the inhibitory shift that will occur at time $t_{0}+d$. Obtaining this expected value is not trivial, so this will be done by a combination of analytical and numerical methods in two stages. The first stage provides a very simple version of $\varDelta _{\mathrm{eq}}$, which assumes no inputs between times $t_{0}$ and $t_{0}+d$. The second stage goes through the trouble of finding an approximation to $E [\varDelta _{\mathrm{inh}}(\phi(t_{0}+d)) ]$ given Poisson inputs, yielding a better version of $\varDelta _{\mathrm{eq}}$, which approximates Eq. (20). As it turns out, our work is not ready here; this is only the second of five $\varDelta _{\mathrm{eq}}$ versions in this subsection.

The third version of $\varDelta _{\mathrm{eq}}$ is required because the equivalent PRC in Eq. (20) is insufficient to ensure that on average $\phi^{+}(t_{0}+d) = \phi_{\mathrm{eq}}(t_{0}+d)$. Although Eq. (20) ensures that the phase shifts resulting from the inputs at $t_{0}$ and at $t_{0}+d$ in $O_{\mathrm{ei}}$ are on average the same as the phase shift at $t_{0}$ in $O_{\mathrm{eq}}$, we still have to consider the phase shifts from inputs between $t_{0}$ and $t_{0}+d$. During this period the inputs will shift the phase of $O_{\mathrm{ei}}$ according to $\varDelta _{\mathrm{exc}}$ and $\varDelta _{\mathrm{inh}}$; on the other hand $O_{\mathrm{eq}}$ will shift its phase according to $\varDelta _{\mathrm{eq}}$ at the time of excitatory inputs, resulting in different values for $\phi^{+}(t_{0}+d)$ and $\phi_{\mathrm{eq}}(t_{0}+d)$. An iterative numerical method will thus be introduced to solve this deficiency, resulting in the third version of $\varDelta _{\mathrm{eq}}$.

The fourth version of $\varDelta _{\mathrm{eq}}$ will be the equivalent of the second version (based on Eq. (20)), but for the case of heterogeneous inputs (e.g. many different inputs to the oscillator, each with its own excitatory and inhibitory PRCs). The fifth version of $\varDelta _{\mathrm{eq}}$ will be the analog of the third version for the case of heterogeneous inputs.

We now begin obtaining the five versions of $\varDelta _{\mathrm{eq}}$.

#### First $\varDelta _{\mathrm{eq}}$ Version

Let us first consider the case of an oscillator with a single Poisson input and feedforward inhibition. As before, it should be remembered that all phases are interpreted to be modulo 1. If an excitatory input arrives at time $t_{n}$ when the phase is $\phi_{n}$, then the phase will immediately experience a shift $\varDelta _{\mathrm{exc}}(\phi_{n})$. At time $t_{n} + d$ the phase will be $(\phi_{n} + \varDelta _{\mathrm{exc}}(\phi_{n}) + \omega d)$, where *ω* is the angular frequency of the oscillator. At that moment the inhibitory input will arrive, causing a phase shift $\varDelta _{\mathrm{inh}}(\phi_{n} + \varDelta _{\mathrm{exc}}(\phi_{n}) + \omega d)$. A simple way to define $\varDelta _{\mathrm{eq}}$ for this case is

21 $$ \varDelta _{\mathrm{eq}}(\phi) \equiv \varDelta _{\mathrm{exc}}(\phi) + \varDelta _{\mathrm{inh}}\bigl(\phi+ \varDelta _{\mathrm{exc}}(\phi) + \omega d\bigr). $$

This constitutes the first of the five versions for the equivalent PRC. One difficulty that quickly becomes apparent with it, is that in the time interval between $t_{n}$ and $t_{n} + d$ there will usually be other inputs arriving at the oscillator, so that the phase when the inhibitory input arrives will generally not be $(\phi+ \varDelta _{\mathrm{exc}}(\phi) + \omega d)$. Indeed, this approach only produces reasonable results when inputs are unlikely to arrive between $t_{n}$ and $t_{n}+d$, which could happen when the value of *d* is very small.

#### Second $\varDelta _{\mathrm{eq}}$ Version

One way to improve our equivalent PRC is to substitute $\varDelta _{\mathrm{inh}}(\phi+ \varDelta _{\mathrm{exc}}(\phi) + \omega d)$ by the expected value of the inhibitory shift given the phase when the excitatory shift happened, as was done in Eq. (20). Let $\theta(t)$ be the function that gives the phase of the oscillator at time *t*. Assume that an excitatory input arrives at time $t_{0}$, when the phase is $\phi_{0}$, meaning $\phi_{0} = \theta(t_{0})$. Furthermore, assume that between the times $t_{0}$ and $t_{0}+d$ there arrive $k_{e}$ excitatory inputs at the times $t_{j}^{e}$, $j = 1,\dots,k_{e}$; and $k_{i}$ inhibitory inputs at the times $t_{m}^{i}$, $m = 1,\dots,k_{i}$. For these particular initial phase and inputs define the phase deviation as

22 $$ D = \sum_{j=1}^{k_{e}} \varDelta _{\mathrm{exc}} \bigl(\theta\bigl(t_{j}^{e}\bigr)\bigr) + \sum _{m=1}^{k_{i}} \varDelta _{\mathrm{inh}}\bigl(\theta \bigl(t_{m}^{i}\bigr)\bigr). $$

*D* is a random variable that tells us how much the phase will change due to inputs during the interval between $t_{0}$ and $t_{0}+d$. For notational convenience let us define $a \equiv \phi + \varDelta _{\mathrm{exc}}(\phi)$, and $b \equiv \phi + \varDelta _{\mathrm{exc}}(\phi) + \omega d$. Our goal is to calculate the expected value of $\varDelta _{\mathrm{inh}}(\phi + \varDelta _{\mathrm{exc}}(\phi) + \omega d + D)$, which is denoted by $E(\varDelta _{\mathrm{inh}}(b + D)|\phi)$. This notation indicates the expected value of the inhibitory shift given that the excitatory shift happened when the phase was *ϕ*. The equivalent PRC can then be defined as:

23 $$ \varDelta _{\mathrm{eq}}(\phi) \equiv \varDelta _{\mathrm{exc}}(\phi) + E\bigl( \varDelta _{\mathrm{inh}}(b + D)|\phi\bigr). $$

This is the second version of the equivalent PRC in this subsection. The following paragraphs deal with finding a practical way to calculate $E(\varDelta _{\mathrm{inh}}(b + D)|\phi)$, culminating with Eq. (27), which can be used in conjunction to Eq. (24) to obtain an approximation for this expected value.

To calculate $E(\varDelta _{\mathrm{inh}}(b + D)|\phi)$, we can start by calculating this expected value when we know exactly how many excitatory and inhibitory inputs arrived during the delay period. Assume that the inputs are independent Poisson point processes, with rate $r_{e}$ for the excitatory ones, and rate $r_{i}$ for the inhibitory ones. Using the PDF for the Poisson distribution we can obtain

24 $$ \begin{aligned}[b] E\bigl(\varDelta _{\mathrm{inh}}(b + D)|\phi\bigr) = {}&\sum _{k_{e}=0}^{\infty}\sum_{k_{i}=0}^{\infty}\biggl[ \frac{(r_{e} d)^{k_{e}}}{k_{e}!} \mathrm{e}^{-r_{e} d} \biggr] \biggl[ \frac{(r_{i} d)^{k_{i}}}{k_{i}!} \mathrm{e}^{-r_{i} d} \biggr]\\ &{}\times E\bigl(\varDelta _{\mathrm{inh}}(b+D)| \phi,k_{e},k_{i}\bigr), \end{aligned} $$

where $E(\varDelta _{\mathrm{inh}}(b+D)|\phi,k_{e},k_{i})$ is the expected value of the inhibitory shift given that there were $k_{e}$ excitatory and $k_{i}$ inhibitory inputs during the delay period, making no assumptions about the order in which they arrived. The assumption of independence between excitatory and inhibitory inputs is based on the fact that we are restricted to a time interval of length *d*, during which none of the inhibitory inputs is the result of feedforward inhibition from one of the excitatory inputs. Notice that the first two factors decay exponentially, so in practice it is only necessary to use a moderate number of terms.

The strategy to obtain $E(\varDelta _{\mathrm{inh}}(b+D)|\phi,k_{e},k_{i})$ is to first find the PDF of *D*, so we can then find the expected value through integration. This calculation can become involved, so I will first focus on the simpler case when there is only a single excitatory input and no inhibitory inputs during the delay interval. Under these circumstances, if the initial excitatory stimulus arrived at phase *ϕ*, the PDF of *D* is denoted by $p(D|\phi,k_{e}=1,k_{i}=0)$. What follows is some formal reasoning to arrive at an expression for $E(\varDelta _{\mathrm{inh}}(b + D)|a,k_{e}=1,k_{i}=0)$. The reader may just go directly to Eq. (25), which is intuitive enough.

Let $\mathcal{B}$ denote the Borel sets in the interval $[0,a_{\mathrm{exc}}]$, where $a_{\mathrm{exc}}$ is the largest value on the range of $\varDelta _{\mathrm{exc}}$, and define a function $I^{e}:\mathcal{B} \rightarrow \mathcal{B}$ that maps each set $A \in \mathcal{B}$ to its preimage under $\varDelta _{\mathrm{exc}}$. Given the fact that there was only a single input coming from the Poisson process in the phase interval $[a,b]$, I make the assumption that the input could have arrived with equal probability at any phase between *a* and *b*. This implies that for an interval *H* in $[0,a_{\mathrm{exc}}]$ the probability of $D \in H$ is given by $\lambda(I^{e}(H)\cap [a,b])/\lambda([a,b])$, where *λ* is the standard Lebesgue measure for the real numbers. Put into words, the probability that the phase advance *D* produced by the sole excitatory input is in the set *H* is the probability of the phases that are mapped into *H* by $\varDelta _{\mathrm{exc}}$.

Notice that $\lambda([a,b]) = \omega d$. If we define a measure $\mu(H) = \lambda(I^{e}(H)\cap [a,b])/\omega d$, then the PDF of *D* will be the Radon–Nikodym derivative of *μ* with respect to *λ*. A practical way to calculate this PDF starts by partitioning the interval $[0,a_{\mathrm{exc}}]$ into subintervals were $\varDelta _{\mathrm{exc}}$ is invertible or constant, which should be possible for any reasonable PRC. If $\varDelta _{\mathrm{exc}}$ is invertible in the interval $[\alpha,\beta]$, and $x = \varDelta _{\mathrm{exc}}(\alpha)$, $y=\varDelta _{\mathrm{exc}}(\beta)$, then

$$\begin{aligned} \mu\bigl([x,y]\bigr) &= \lambda\bigl(I^{e}\bigl([x,y]\cap [a,b]\bigr) \bigr)/\omega d\\ & = \frac{|\varDelta _{\mathrm{exc}}^{-1}(y) -\varDelta _{\mathrm{exc}}^{-1}(x)|}{\omega d} = \frac{1}{\omega d} \int _{[\varDelta _{\mathrm{exc}}^{-1}(y),\varDelta _{\mathrm{exc}}^{-1}(x)]}\,d \lambda. \end{aligned}$$

The change of variables formula shows that

$$\begin{aligned} \int_{[\varDelta _{\mathrm{exc}}^{-1}(y),\varDelta _{\mathrm{exc}}^{-1}(x)]}\,d \lambda & = \int_{x}^{y} \biggl\lvert \frac{d}{ds} \varDelta _{\mathrm{exc}}^{-1}(s) \biggr\rvert \,ds = \int_{x}^{y} \frac{1}{|\varDelta _{\mathrm{exc}}'(\varDelta _{\mathrm{exc}}^{-1}(s))|}\,ds. \end{aligned}$$

If $\varDelta _{\mathrm{exc}}' \neq 0$ in $[\alpha,\beta]$ in sets other than those of zero measure we can write

$$ P\bigl(D \in [x,y]|\phi,k_{e}=1,k_{i}=0\bigr) = \frac{1}{\omega d} \int_{x}^{y} \bigl\vert \varDelta _{\mathrm{exc}}'\bigl(\varDelta _{\mathrm{exc}}^{-1}(s) \bigr)\bigr\vert ^{-1}\,ds, $$

which means that $|\varDelta _{\mathrm{exc}}'(\varDelta _{\mathrm{exc}}^{-1}(s))|^{-1}/\omega d$ is the PDF of *D*. If we have an interval where $\varDelta _{\mathrm{exc}}$ is equal to a constant *c*, the integral becomes undefined due to a zero in the denominator. In this case we have $P(D = c|\phi,k_{e}=1,k_{i}=0) = \mu(c)$, and finding the expected value of the inhibition is trivial. For invertible intervals we can now write the expected value of the inhibition as

$$ E\bigl(\varDelta _{\mathrm{inh}}(\beta + D)|\alpha,k_{e}=1,k_{i}=0 \bigr) = \frac{1}{\omega d} \int_{x}^{y} \bigl\vert \varDelta _{\mathrm{exc}}'\bigl(\varDelta _{\mathrm{exc}}^{-1}(s) \bigr)\bigr\vert ^{-1} \varDelta _{\mathrm{inh}}(\beta + s)\,ds. $$

Using a change of variables this becomes the more intuitive formula

$$ E\bigl(\varDelta _{\mathrm{inh}}(\beta + D)|\alpha,k_{e}=1,k_{i}=0 \bigr) = \frac{1}{\omega d} \int_{\alpha}^{\beta} \varDelta _{\mathrm{inh}}\bigl(\beta + \varDelta _{\mathrm{exc}}(s)\bigr)\,ds. $$

Notice that after the change of variables the integral can handle the case where $\varDelta _{\mathrm{exc}}$ is constant. Considering that the value of an integral on the interval $[a,b]$ is the sum of the integral on the subintervals $[\alpha,\beta]$ where $\varDelta _{\mathrm{exc}}$ is invertible or constant, we can now write

25 $$ E\bigl(\varDelta _{\mathrm{inh}}(b + D)|a,k_{e}=1,k_{i}=0\bigr) = \frac{1}{\omega d} \int_{a}^{b} \varDelta _{\mathrm{inh}}\bigl(b + \varDelta _{\mathrm{exc}}(s)\bigr)\,ds. $$

In a similar manner it can be shown that

$$ E\bigl(\varDelta _{\mathrm{inh}}(b + D)|a,k_{e}=0,k_{i}=1\bigr) = \frac{1}{\omega d} \int_{a}^{b} \varDelta _{\mathrm{inh}}\bigl(b + \varDelta _{\mathrm{inh}}(s)\bigr)\,ds. $$

The complexity of these equations increases once we have more than one input, and once we have both excitatory and inhibitory inputs, because the order in which they arrive is important. In this case the expected value for the inhibition comes from averaging over all the possible phases when the first and second stimuli could have arrived, and over the possible orders for the arrival of stimuli. To illustrate this, let us look at the formula for $k_{e} =1$, $k_{i}= 1$

$$\begin{aligned} &E\bigl(\varDelta _{\mathrm{inh}}(b + D)|\phi,k_{e}=1,k_{i}=1 \bigr) \\ &\quad = \frac{1}{2 \omega d} \biggl[ \int_{a}^{b} \frac{1}{b - \psi_{1}} \int_{\psi_{1} + \varDelta _{\mathrm{exc}}(\psi_{1})}^{b + \varDelta _{\mathrm{exc}}(\psi_{1})} \varDelta _{\mathrm{inh}}\bigl(b + \varDelta _{\mathrm{exc}}(\psi_{1}) + \varDelta _{\mathrm{inh}}(\psi_{2})\bigr)\,d \psi_{2} \,d \psi_{1} \\ &{}\qquad + \int_{a}^{b} \frac{1}{b - \psi_{1}} \int _{\psi_{1} + \varDelta _{\mathrm{inh}}(\psi_{1})}^{b + \varDelta _{\mathrm{inh}}(\psi_{1})} \varDelta _{\mathrm{inh}}\bigl(b + \varDelta _{\mathrm{inh}}(\psi_{1}) + \varDelta _{\mathrm{exc}}( \psi_{2})\bigr)\,d \psi_{2} \,d \psi_{1} \biggr]. \end{aligned}$$

Intuitively, the integration variable $\psi_{1}$ stands for the phase when the first input arrived, and $\psi_{2}$ for the phase when the second input arrived. The first pair of nested integrals are for the case when the excitatory input happened first, and the second ones for the case when the inhibitory input was the first to arrive. The innermost integrals obtain the average inhibition given that the first stimulus arrived at phase $\psi_{1}$, and in the limit when $\psi_{1} \rightarrow b$, they converge to the $\varDelta _{\mathrm{inh}}$ expression with $\psi_{1}$ substituted by *b*, and $\psi_{2}$ substituted by $b + \varDelta _{\mathrm{exc}/\mathrm{inh}}(b)$.

In order to write the formula for the case with arbitrary values for $k_{e}$ and $k_{i}$ some preliminary definitions are required. Notice that if we have $k_{e}$ excitatory and $k_{i}$ inhibitory inputs, there are $C_{k_{e}}^{k_{e}+k_{i}}$ different input sequences according to whether the *j*th input was excitatory or inhibitory. Let us index those sequences and denote them by $\sigma_{i}$. In other words, we create $C_{k_{e}}^{k_{e}+k_{i}}$ functions $\sigma_{i}:\{1,2,\dots,k_{i} + k_{e}\} \rightarrow \{-1,1\}$, defined by

$$ \sigma_{i}(j) = \textstyle\begin{cases} 1, & \mbox{if the }j \mbox{th element of the }i \mbox{th sequence is excitatory}; \\ -1, & \mbox{if the }j \mbox{th element of the }i \mbox{th sequence is inhibitory}. \end{cases} $$

Now define the function $\varDelta _{\mathrm{mix}}:[0,1]\times\{1,\dots,C_{k_{e}}^{k_{e}+k_{i}}\}\times\{1,\dots,k_{e}+k_{i}\} \rightarrow R$ (where *R* stands for the real numbers) by

$$ \varDelta _{\mathrm{mix}}(\phi,i,j) = \textstyle\begin{cases} \varDelta _{\mathrm{exc}}(\phi), & \mbox{if } \sigma_{i}(j) = 1; \\ \varDelta _{\mathrm{inh}}(\phi), & \mbox{if } \sigma_{i}(j) = -1. \end{cases} $$

The general formula can now be written as

26 $$\begin{aligned} & E\bigl(\varDelta _{\mathrm{inh}}(b + D)|\phi,k_{e},k_{i}\bigr) \\ &\quad =\bigl(\omega d C_{k_{e}}^{k_{e}+k_{i}} \bigr)^{-1} \\ &\qquad{} \times\sum _{i=1}^{C_{k_{e}}^{k_{e}+k_{i}}} \Biggl[ \int_{a}^{b} d \psi_{1} \frac{1}{b-\psi_{1}} \int_{\psi_{1} + \varDelta _{\mathrm{mix}}(\psi_{1},i,1)}^{b + \varDelta _{\mathrm{mix}}(\psi_{1},i,1)} \,d \psi_{2} \frac{1}{b + \varDelta _{\mathrm{mix}}(\psi_{1},i,1) -\psi_{2}} \cdots \\ &\qquad{}\times \int_{\psi_{(k_{e}+k_{i}-1)} + \varDelta _{\mathrm{mix}}(\psi_{(k_{e}+k_{i}-1)},i,k_{e}+k_{i}-1)} ^{b + \sum_{m=1}^{k_{e}+k_{i}-1}\varDelta _{\mathrm{mix}}(\psi_{m},i,m)}\,d \psi_{(k_{e}+k_{i})} \\ &\qquad{}\times \varDelta _{\mathrm{inh}} \Biggl(b + \sum_{j=1}^{k_{e}+k_{i}} \varDelta _{\mathrm{mix}}(\psi_{j},i,j) \Biggr) \Biggr]. \end{aligned}$$

For this equation I have used the convention of writing the differential sign next to its corresponding integration sign.

Although Eq. (26) expresses the expected inhibition values that we want to calculate, its complexity makes it virtually useless for practical purposes. Fortunately, a simple assumption can simplify this expression. Assume that for each input sequence, the inputs happen at regular time intervals (the time periods between any two inputs are equal). It is simple to calculate the expected value of the inhibition for this case. If we have $K = k_{e} + k_{i}$ inputs, define $\gamma = \omega d/(K+1)$, and for $i = 1,\dots,C_{k_{e}}^{k_{e}+k_{i}}$ let

$$\begin{aligned} \theta_{0}^{i} =& a, \\ \theta_{1}^{i} =& \theta_{0}^{i} + \gamma + \varDelta _{\mathrm{mix}}\bigl(\theta_{0}^{i} + \gamma,i,1\bigr), \\ \theta_{2}^{i} =& \theta_{1}^{i} + \gamma + \varDelta _{\mathrm{mix}}\bigl(\theta_{1}^{i} + \gamma,i,2\bigr), \\ \vdots& \\ \theta_{K}^{i} =& \theta_{K-1}^{i} + \gamma + \varDelta _{\mathrm{mix}}\bigl(\theta_{K-1}^{i} + \gamma,i,K\bigr). \end{aligned}$$

We then have

27 $$ E\bigl(\varDelta _{\mathrm{inh}}(b+D)|\phi,k_{e},k_{i},\mbox{RT} \bigr) = \frac{1}{C_{k_{e}}^{k_{e}+k_{i}}} \sum_{i=1}^{C_{k_{e}}^{k_{e}+k_{i}}} \varDelta _{\mathrm{inh}}\bigl(\theta_{K}^{i} + \gamma\bigr), $$

where RT stands for “Regular Times,” meaning that the inputs arrive at regular time intervals. For a smooth function $\varDelta _{\mathrm{inh}}$ and relatively small values of the feedforward delay *d* we will have

$$ E\bigl(\varDelta _{\mathrm{inh}}(b+D)\bigl\vert \phi,k_{e},k_{i}, \mbox{RT}\bigr) \approx E\bigl(\varDelta _{\mathrm{inh}}(b+D)\bigr\vert \phi,k_{e},k_{i}\bigr). $$

#### Third $\varDelta _{\mathrm{eq}}$ Version

Even if we now can obtain a good approximation to the expected phase shift caused by feedforward inhibition, the equivalent PRC from Eq. (23) may still not achieve the goal of reaching, on average, the same phase as the oscillator with two PRCs after the feedforward delay. To make this explicit, assume that the function $\theta(t)$ provides the phase of the oscillator with feedforward inhibition at time *t*, just as it is described for Eq. (22), and let $\theta_{\mathrm{eq}}(t)$ give the phase of an oscillator using the corresponding equivalent PRC from Eq. (23) when the input times are the same. Using an equivalent PRC instead of $\varDelta _{\mathrm{exc}}$ and $\varDelta _{\mathrm{inh}}$ causes the phase deviation of Eq. (22) to become

$$ D_{\mathrm{eq}} = \sum_{j=1}^{k_{e}} \varDelta _{\mathrm{eq}}\bigl(\theta_{\mathrm{eq}}\bigl(t_{j}^{e} \bigr)\bigr). $$

In general, $D \neq D_{\mathrm{eq}}$ during the delay period; we can calculate the expected phase difference that this will cause right after the feedforward delay. If an initial excitatory input arrives at time *t* when the phase is *ϕ*, the expected value of the phase for the oscillator with two PRCs at time $t+d$ right after the feedforward inhibition is

$$ b + E\bigl(\varDelta _{\mathrm{inh}}(b+D)|\phi\bigr) + E(D|\phi). $$

On the other hand, the expected value of the phase for the oscillator with one PRC at time $t+d$ is

$$ b + E\bigl(\varDelta _{\mathrm{inh}}(b+D)|\phi\bigr) + E(D_{\mathrm{eq}}|\phi). $$

Subtracting the previous two expressions gives us the expected value of the phase difference between the two oscillators at time $t+d$ given that there was an excitatory input at time *t* when the phase was *ϕ*, denoted by $\xi(\phi)$:

28 $$ \xi(\phi) = E(D|\phi) - E(D_{\mathrm{eq}}|\phi). $$

If we are capable of calculating $E(D|\phi)$ and $E(D_{\mathrm{eq}}|\phi)$, then we can use $\xi(\phi)$ in order to create an equivalent PRC that produces a smaller value of $\xi(\phi)$. A simple algorithm for doing this is as follows. Let *M* be an integer, and *ϵ* a small real number. Define $\varDelta _{\mathrm{eq}}^{(0)}$ as the equivalent PRC from Eq. (23), and $D_{\mathrm{eq}}^{0}$ as its corresponding phase deviation.

$$\begin{aligned} &\textbf{for } i = 1 \mbox{ to } M \textbf{ do}\\ & \quad \xi^{(i)}(\phi) = E(D|\phi) - E(D_{\mathrm{eq}}^{(i-1)}|\phi)\\ & \quad \varDelta _{\mathrm{eq}}^{(i)}(\phi) = \varDelta _{\mathrm{eq}}^{(i-1)}(\phi) + \epsilon \xi^{(i)}(\phi) \\ & \textbf{end for} \end{aligned}$$

At each step in this algorithm the functions $\xi^{(i)}$ and $\varDelta _{\mathrm{eq}}^{(i)}$ are calculated for all the values of *ϕ*, so it is similar to gradient descent performed for a whole function. The resulting PRC $\varDelta _{\mathrm{eq}}^{(M)}$ constitutes the third version of an equivalent PRC we have obtained, and can already provide very good results for oscillators with a single input, or with homogeneous excitatory and inhibitory PRCs.

#### Fourth $\varDelta _{\mathrm{eq}}$ Version

The fourth version of an equivalent PRC that I will obtain extends the second version to the case when there are heterogeneous inputs. The reason why the approach taken so far to obtain $\varDelta _{\mathrm{eq}}$ may fail when we consider several types of inputs, each with its own excitatory and inhibitory PRCs, is that when Eq. (27) is obtained the phase is assumed to advance at a steady rate between inputs (the phase would advance an amount *γ* between inputs). If there is only one type of input this is justified, since the oscillator has a constant angular frequency. When we have different types of inputs we consider each one separately, so even if inputs of one type arrive at regular intervals in time, the phase will be shifted between consecutive times by inputs of other types. This will become explicit in the following calculation.

Consider an oscillator with feedforward inhibition that receives $N_{\mathrm{syn}}$ different inputs. We consider that for each input there are two “synapses,” one excitatory and one inhibitory, characterized by the PRCs $\varDelta _{\mathrm{exc}}^{i}$, and $\varDelta _{\mathrm{inh}}^{i}$ for the *i*th input. We need to obtain $N_{\mathrm{syn}}$ equivalent PRCs, with $\varDelta _{\mathrm{eq}}^{i}$ being used to replace $\varDelta _{\mathrm{exc}}^{i}$ and $\varDelta _{\mathrm{inh}}^{i}$. The goal is therefore to obtain a version of Eq. (27) that works for each *i*th input individually. In order to model how the oscillator’s phase changes between repetitions of the *i*th input I will use the concept of variable phase velocity, which will be explained next.

Let us say an oscillator has a non-constant phase PDF $\rho(\phi)$. We can think that this oscillator’s phase has a constant rate of change $d\phi/dt = \omega$, but its inputs reshape the phase PDF so it becomes $\rho(\phi)$. Alternatively, we could image that the oscillator receives no inputs, but instead has a phase whose velocity $d\phi/dt = \omega(t)$ is changing over time so that $\rho(\phi)$ is produced. The idea to be introduced here is to use this oscillator with no inputs and variable phase velocity in order to model the oscillator with constant phase velocity and random inputs.

Let *T* denote the period that we will assign to our oscillator. Assume that at time $t=0$ the oscillator has phase 0, and denote the time it will take to reach phase $\phi \in [0,1]$ by $\tau(\phi)$. It is easy to show that when $\tau(\phi) = T \int_{0}^{\phi}\rho(\psi)\,d\psi$ the phase PDF of the oscillator is $\rho(\phi)$. Since $\rho > 0$, $\tau(\phi)$ is a monotone increasing function, which implies that it will be invertible on $[0,1]$. Let $\mathcal{P}$ denote this inverse. The function $\mathcal{P}$ provides the phase as a function of the time since phase 0 was crossed. We consider the argument of $\mathcal{P}$ to be modulo *T*, so it is always in the interval $[0,T]$. We are now ready to create a version of Eq. (27) for the case of heterogeneous inputs.

Let $\rho(\phi)$ be the phase of the oscillator with feedforward inhibition and heterogeneous PRCs. Assume that during the feedforward delay period the synapses for input *j* receive $k_{e}^{j}$ excitatory inputs, and $k_{i}^{j}$ inhibitory inputs. Let $K^{j} = k_{e}^{j} + k_{i}^{j}$, $\tau_{j} = \frac{d}{K^{j} + 1}$. We can now set

$$\begin{aligned} \theta_{0}^{i,j} =& a, \\ \theta_{1}^{i,j} =& \mathcal{P}\bigl(\tau\bigl( \theta_{0}^{i,j}\bigr) + \tau_{j}\bigr) + \varDelta _{\mathrm{mix}} \bigl(\mathcal{P}\bigl(\tau\bigl(\theta_{0}^{i,j} \bigr) + \tau_{j}\bigr),i,1 \bigr), \\ \theta_{2}^{i,j} =& \mathcal{P}\bigl(\tau\bigl( \theta_{1}^{i,j}\bigr) + \tau_{j}\bigr) + \varDelta _{\mathrm{mix}} \bigl(\mathcal{P}\bigl(\tau\bigl(\theta_{1}^{i,j} \bigr) + \tau_{j}\bigr),i,2 \bigr), \\ \vdots& \\ \theta_{K}^{i,j} =& \mathcal{P}\bigl(\tau\bigl( \theta_{K-1}^{i,j}\bigr) + \tau_{j}\bigr) + \varDelta _{\mathrm{mix}} \bigl(\mathcal{P}\bigl(\tau\bigl(\theta_{K-1}^{i,j} \bigr) + \tau_{j}\bigr),i,K\bigr) ; \end{aligned}$$

and also

$$ E\bigl(\varDelta _{\mathrm{inh}}^{j}(b+D)|\phi,k_{e}^{j},k_{i}^{j}, \mbox{RT}\bigr)^{*} = \bigl(C_{k_{e}^{j}}^{k_{e}^{j}+k_{i}^{j}} \bigr)^{-1} \sum_{i=1}^{C_{k_{e}^{j}}^{k_{e}^{j}+k_{i}^{j}}} \varDelta _{\mathrm{inh}}^{j} \bigl(\mathcal{P}\bigl(\tau\bigl(\theta_{K}^{i,j}\bigr) + \tau_{j}\bigr) \bigr), $$

where the ^∗^ symbol next to the expected value denotes the fact that we used the variable phase velocity approach. The definition of the phase deviation *D* in this equation reflects the case of heterogeneous synapses:

$$ D = \sum_{j=1}^{N_{\mathrm{syn}}} \Biggl( \sum _{l=1}^{k_{e}^{j}} \varDelta _{\mathrm{exc}}^{j}\bigl( \theta\bigl(t_{l,j}^{e}\bigr)\bigr) + \sum _{m=1}^{k_{i}^{j}} \varDelta _{\mathrm{inh}}^{j}\bigl( \theta\bigl(t_{m,j}^{i}\bigr)\bigr) \Biggr). $$

Obtaining the equivalent PRC proceeds as before. Let $b_{j} = \phi + \varDelta _{\mathrm{exc}}^{j}(\phi) + \omega d$; we now have

$$\begin{aligned} E\bigl(\varDelta _{\mathrm{inh}}^{j}(b_{j} + D)|\phi\bigr) \approx& \sum_{k_{e}^{j}=0}^{\infty}\sum _{k_{i}^{j}=0}^{\infty}\biggl[ \frac{(r_{e}^{j} d)^{k_{e}^{j}}}{k_{e}^{j}!} \mathrm{e}^{-r_{e}^{j} d} \biggr] \biggl[ \frac{(r_{i}^{j} d)^{k_{i}^{j}}}{k_{i}^{j}!} \mathrm{e}^{-r_{i}^{j} d} \biggr] \\ &{}\times E\bigl(\varDelta _{\mathrm{inh}}^{j}(b_{j}+D)| \phi,k_{e}^{j},k_{i}^{j},RT\bigr)^{*}, \end{aligned}$$

where $r_{e}^{j}$ and $r_{i}^{j}$ are the excitatory and inhibitory firing rates of the *j*th input. Since we are considering the case of feedforward inhibition, we will have $r_{e}^{j} = r_{i}^{j}$. The fourth version of the equivalent PRC is

29 $$ \varDelta _{\mathrm{eq}}^{j}(\phi) = \varDelta _{\mathrm{exc}}^{j}( \phi) + E\bigl(\varDelta _{\mathrm{inh}}^{j}(b_{j} + D)|\phi \bigr). $$

#### Fifth $\varDelta _{\mathrm{eq}}$ Version

As would be expected, the fourth version of $\varDelta _{\mathrm{eq}}$ has the same limitations as the second version, and those limitations can be surmounted using the same approach that led from the second to the third equivalent PRC. The phase deviation when using the equivalent synapses from the fourth PRC is

$$ D_{\mathrm{eq}} = \sum_{j=1}^{N_{\mathrm{syn}}} \sum _{l=1}^{k_{e}^{j}} \varDelta _{\mathrm{eq}}^{j} \bigl(\theta_{\mathrm{eq}}\bigl(t_{l,j}^{e}\bigr)\bigr). $$

If at time *t* an excitatory input is received by the *j*th synapse, the expected phase for the oscillator with feedforward inhibition and heterogeneous synapses at time $t+d$ is

$$b_{j} + E(D|\phi,j) + E\bigl[\varDelta _{\mathrm{inh}}^{j}(b_{j} + D)|\phi,j\bigr]. $$

The notation $E(D|\phi,j)$ indicates the assumption that a shift of magnitude $\varDelta _{\mathrm{exc}}^{j}(\phi)$ happens at time *t*. In the case of the oscillator with the equivalent PRCs of Eq. (29), the expected phase at time $t+d$ is

$$b_{j} + E(D_{\mathrm{eq}}|\phi,j) + E\bigl[\varDelta _{\mathrm{inh}}^{j}(b_{j} + D)|\phi,j\bigr]. $$

Similarly to Eq. (28), the difference in phase at time $t+d$ is

$$ \xi_{j}(\phi) = E(D|\phi,j) - E(D_{\mathrm{eq}}|\phi,j). $$

Let the functions $\varDelta _{\mathrm{eq}}^{j,(0)}$ come from Eq. (29), for $j = 1,\dots,N_{\mathrm{syn}}$. The procedure to obtain the fifth version of the equivalent PRC is as follows:

$$\begin{aligned} &\textbf{for } i = 1\mbox{ to }M\textbf{ do} \\ &\quad \textbf{for } j = 1\mbox{ to }N_{\mathrm{syn}}\textbf{ do} \\ & \qquad \xi^{(i)}_{j}(\phi) = E(D|\phi,j) - E(D_{\mathrm{eq}}^{(i-1)}|\phi,j)\\ & \qquad \varDelta _{\mathrm{eq}}^{j,(i)}(\phi) = \varDelta _{\mathrm{eq}}^{j,(i-1)}(\phi) + \epsilon \xi^{j,(i)}_{j}(\phi) \\ & \quad \textbf{end for} \\ & \textbf{end for} \end{aligned}$$

The value $E(D_{\mathrm{eq}}^{(i-1)}|\phi,j)$ is the expected phase deviation calculated using the equivalent PRCs $\varDelta _{\mathrm{eq}}^{j,(i-1)}$. This calculation can be time consuming depending on the method used, so the algorithm can be modified by dividing the $N_{\mathrm{syn}}$ equivalent PRCs into $N_{\mathrm{batch}}$ batches. Then for each batch we use the same $\xi^{j,(i)}_{j}$ in order to update the PRCs.

#### Computational Simulations

**Fig. 3:**
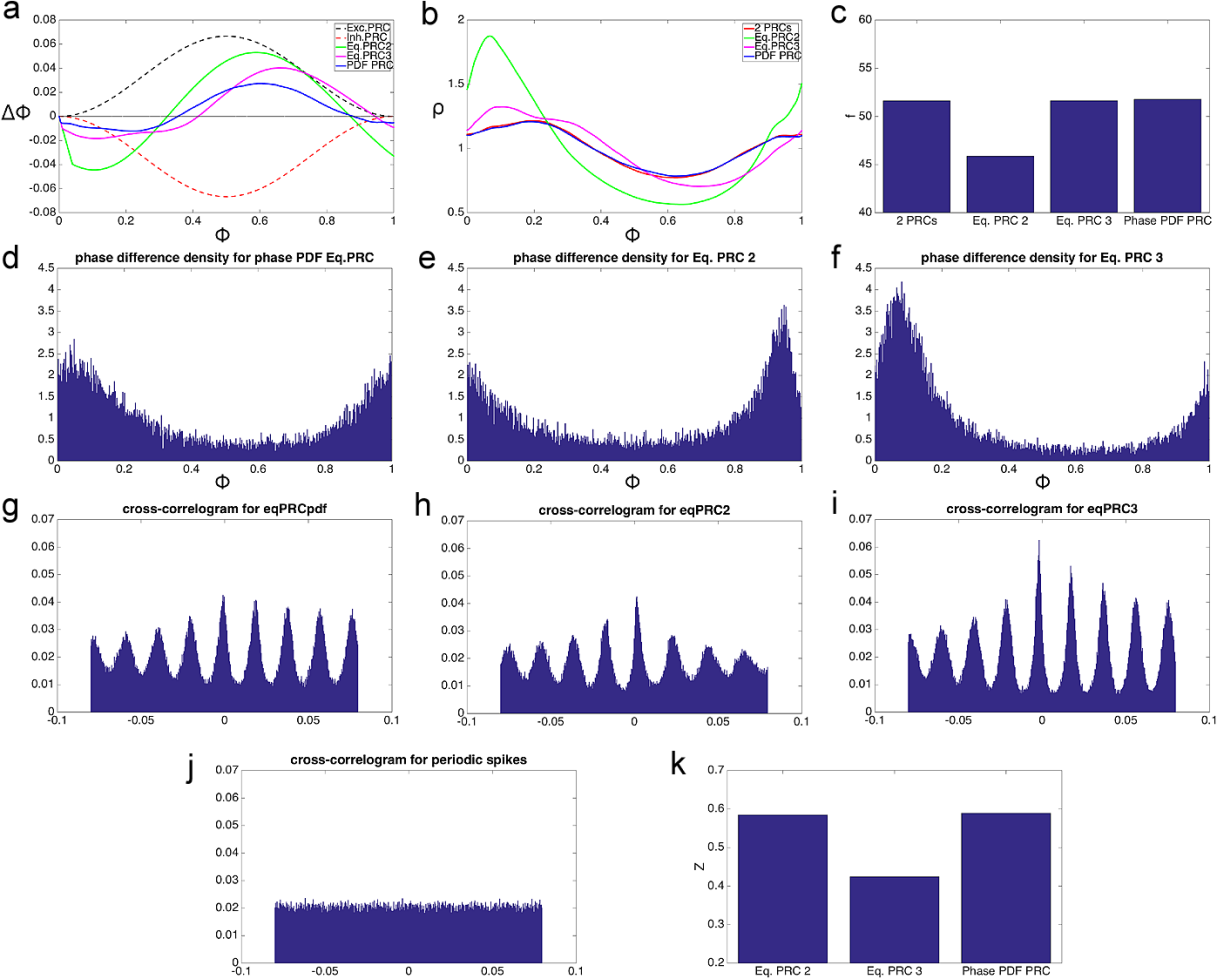
Comparison of the equivalent PRCs for homogeneous inputs. Result of a simulation using four types of oscillators. The first one has both excitatory and inhibitory PRCs of amplitudes $a_{\mathrm{exc}}=2/30$ and $a_{\mathrm{inh}}=2/30$, respectively, with a feedforward delay of 5 ms. The second one has the equivalent PRC *Δ* from the previous subsection (Eqs. (15) and (19)), labeled “phase PDF Eq. PRC.” The third one has the second equivalent PRC from this subsection (Eq. PRC 2). The fourth one has the third equivalent PRC from this subsection (Eq. PRC 3). The three oscillators with the equivalent PRCs only get stimulated at the time of the excitatory inputs, which have a rate of 600 Hz. The other oscillator experiences feedforward inhibition. **a**: Shape of the PRCs. *The dashed black and red lines* show $\varDelta _{\mathrm{exc}}$ and $\varDelta _{\mathrm{inh}}$, respectively. *The blue line* corresponds to the PRC *Δ* from the previous subsection. *The green line* corresponds to Eq. PRC 2, and *the magenta line* to Eq. PRC3. **b**: Phase PDFs for the four oscillators. *The red line* (partially occluded by *the blue line*) shows the phase PDF for the oscillator with feedforward inhibition. *The blue line* corresponds to the oscillator with Eq. PRC *Δ* from the previous subsection. *The green line* is from the oscillator with Eq. PRC 2, and *the magenta line* for the oscillator with Eq. PRC 3. **c**: Average firing rate for the four oscillators. **d**–**f**: Phase difference density (see text) for the three oscillators with equivalent PRCs. **g**–**i**: Cross-correlograms between the output spike trains of the three oscillators with equivalent PRCs and the output spike train of the oscillator with feedforward inhibition. **j**: Cross-correlogram between the output spike train of the oscillator with feedforward inhibition and a periodic spike train with the same frequency. **k**: Circular variance (see text) for the three oscillators with equivalent PRCs

As before, I use computational simulations in order to validate the approximations obtained in this subsection. First, in Fig. 3 I compare the second and third equivalent PRCs from this subsection with the equivalent PRC *Δ* obtained from Eqs. (15) and (19) in the previous subsection. The performance of the first PRC from this subsection is significantly worse than any of these three and is not shown. We have at this point several methods to obtain an equivalent PRC, and the point of Fig. 3 is to see how those methods measure against each other. To make this comparison, we can simulate an oscillator with feedforward inhibition, and oscillators with the different equivalent PRCs, all of these receiving inputs at the times when the excitatory inputs arrive for the oscillator with feedforward inhibition. We can then see which of the oscillators with an equivalent PRC had the phases and output spike train that best match those of the oscillator with feedforward inhibition.

Panel b of Fig. 3 shows that using *Δ* leads to a much better agreement in phase PDFs than when using the second or third equivalent PRCs. This is not surprising, since *Δ* was defined with this purpose in mind. What is remarkable is that despite this, the third PRC provides a greater coherence in the output spike trains, as will be shown below.

In Fig. 3c we can see that *Δ* and the third equivalent PRC can match the firing rate of the oscillator with two PRCs. In the case of *Δ* this is the result of adjusting one parameter, but in the case of the third Equiv. PRC this is a consequence of the iterative procedure that leads from the second to the third version of $\varDelta _{\mathrm{eq}}$. The inability of the second PRC to match firing rates stems largely from the fact that it doesn’t account for the correct average phase shifts during the feedforward delay period.

We have stated that an ideal result would be for the oscillator with feedforward delay and the oscillator with the equivalent PRC to produce the same output spike trains. We should therefore compare the output spike trains between the two oscillators. A graphical way of doing this is with cross-correlograms, as in Fig. 2c, d. Panels g–i of Fig. 3 show cross-correlograms between the output spike train of the oscillator with feedforward inhibition and the oscillators with equivalent PRCs. In addition, Fig. 3j shows the cross-correlogram of the spike train from the oscillator with feedforward inhibition, and a periodic spike train of the same frequency.

A perhaps simpler way to visualize the coherence between the output spike trains comes from the phase difference density, shown in Fig. 3d–f. To calculate this, each time the oscillator with feedforward inhibition spiked, the phase of the other oscillator was measured. This resulted in a sample of oscillator phases, whose normalized histogram constitutes the phase difference density plots shown in panels d–f. Notice that if the two oscillators always spiked at the same time, then the phased difference density plot would have a single tall and narrow peak at zero, and if the two oscillators spiked at uncorrelated times the plot would be flat. As with the cross-correlogram, it can be seen that the largest peaks are obtained with the third equivalent PRC. A way to quantify this comes from the circular variance measure in panel k.

Circular variance [60] is a procedure used to obtain the variance in a sample whose data points are periodic. In this case, since the phases 0 and 1 are the same (and the phases 0.99 and 0.01 are very close), using the normal procedures for averaging produces unwanted results. To calculate the circular variance we start with a sample of phases $\phi_{i}$, $i = 1,\dots,N$, in this case the phases collected to produce the phase difference density plots. Since the phases are periodic, in order to average them they are represented as complex numbers with unit norm. If the number corresponding to $\phi_{i}$ is $s_{i}$, then $\mathbb{R}\{s_{i}\} = \cos(\phi_{i})$, $\mathbb{I}\{s_{i}\} = \sin(\phi_{i})$, and the average of these numbers is $\bar{s} = \frac{1}{N} \sum_{i=1}^{N} ( \cos(\phi_{i}) + i \sin(\phi_{i}) )$. The circular variance is defined as $Z \equiv 1 - \Vert \bar{s} \Vert $. From this definition it can be seen that smaller values of *Z* indicate a larger degree of coherence, and taller, narrow peaks in the phase difference density plot.

Figure 3k shows that the circular variance is significantly smaller when using the third equivalent PRC. This is results shows, at least numerically, that a close match in the phase PDF of two oscillators does not necessarily entail an optimal match in their output spike trains, even when their firing rates are very close.

**Fig. 4:**
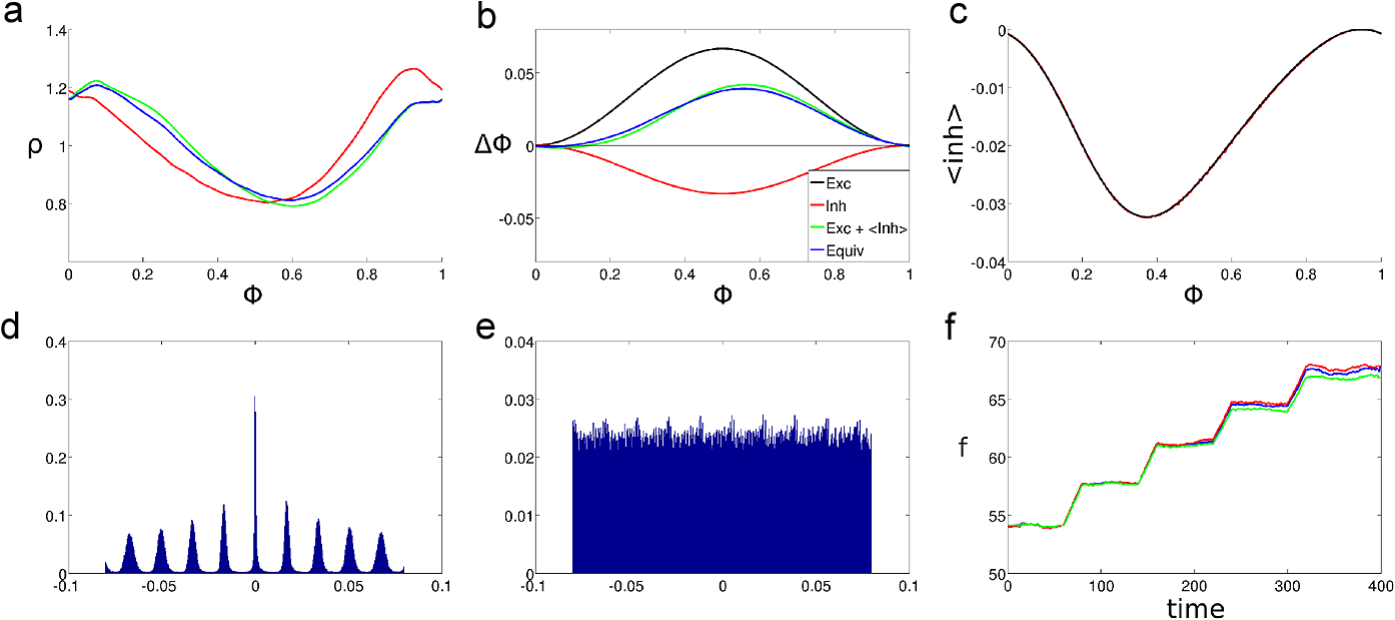
Performance of the second and third equations, PRC versions (homogeneous inputs) with 1 ms delay. These figures compare the results of simulating an oscillator with feedforward inhibition against those of an oscillator with the equivalent PRC when the feedforward delay is 1 ms. The amplitudes for the excitatory and inhibitory PRCs are $a_{\mathrm{exc}} = 2/30$, and $a_{\mathrm{inh}} = 1/30$, respectively. The input rate is 600 Hz. **a**: Stationary phase PDFs for the oscillator with feedforward inhibition (*red*), with the second version of the equivalent PRC (*green*), and with the third version of the equivalent PRC (*blue*). **b**: Phase resetting curves. *Black* = excitatory, *red* = inhibitory, *green* = second Equiv. PRC, *blue* = third Equiv. PRC. **c**: Expected value of the inhibition as a function of the phase when the initial excitatory input arrives. *The red curve* comes from simulating the oscillator with feedforward inhibition, and *the black curve* comes from the approximation in Eq. (27). **d**: Cross-correlogram between the output spike trains of the oscillators with feedforward inhibition and with the third equivalent PRC. *The horizontal axis* indicates time shift, and *the vertical axis* the normalized spike count. Normalization was done by dividing the bin spike counts between the total number of spikes in the output of the oscillator with two PRCs. **e**: Cross-correlogram between the output spike train of the oscillator with feedforward inhibition and a spike train with constant interspike intervals and the same frequency. Normalization was performed as in **d**. **f**: Firing rate response of the oscillators with feedforward inhibition (*red*), the second version of Equiv. PRC (*green*), and the third version of Equiv. PRC (*blue*) to five different levels of input rates. Input rates range from 240 Hz to 1200 Hz in constant increments of 240 Hz

**Fig. 5:**
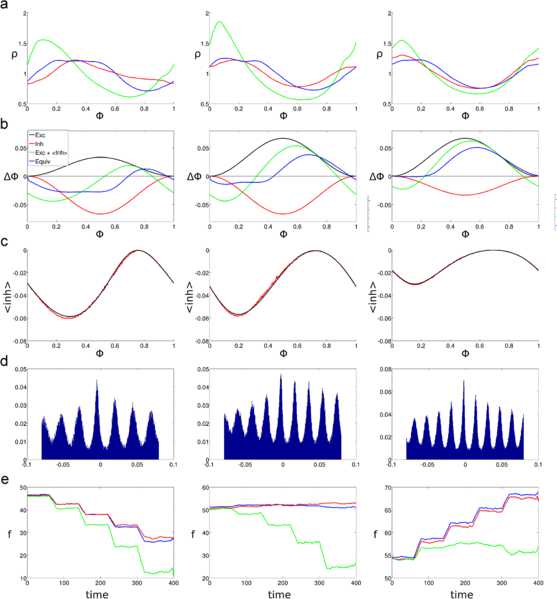
Performance of the second and third equation, PRC versions (homogeneous inputs) with 5 ms delay. Simulations for three different amplitude combinations of $\varDelta _{\mathrm{exc}}$ and $\varDelta _{\mathrm{inh}}$. *The figures on the left* correspond to $a_{\mathrm{exc}} = 1/30$, $a_{\mathrm{inh}} = 2/30$, *figures on the middle* correspond to $a_{\mathrm{exc}} = 2/30$, $a_{\mathrm{inh}} = 2/30$, and *the figures on the right* correspond to $a_{\mathrm{exc}} = 2/30$, $a_{\mathrm{inh}} = 1/30$. The input rate for all simulations in **a**–**d** was $r = 600\mbox{ Hz}$. Other than the input amplitudes, the only difference with the simulation of Fig. 4 is the delay value of 5 ms. **a**: Stationary phase PDFs for the oscillator with feedforward inhibition (*red*), with the second version of the equivalent PRC (*green*), and with the third version of the equivalent PRC (*blue*). **b**: PRCs used in the simulation. $\varDelta _{\mathrm{exc}}$ (*black*), $\varDelta _{\mathrm{inh}}$ (*red*), second version of $\varDelta _{\mathrm{eq}}$ (*green*), third version of $\varDelta _{\mathrm{eq}}$ (*blue*). **c**: Expected value of the inhibition as a function of the phase when the initial excitatory input arrives. *The red curve* comes from simulating the oscillator with feedforward inhibition, and *the black curve* comes from the approximation in Eq. (27). **d**: Cross-correlogram between the output spike trains of the oscillators with feedforward inhibition and with the third equivalent PRC. *The horizontal axis* indicates time shift, and *the vertical axis* the normalized spike count. Normalization was done by dividing the bin spike counts between the total number of spikes in the output of the oscillator with two PRCs. **e**: Firing rate response of the oscillators with feedforward inhibition (*red*), the second version of Equiv. PRC (*green*), and the third version of Equiv. PRC (*blue*) to five different levels of input rates. Input rates range from 240 Hz to 1200 Hz in constant increments of 240 Hz

**Fig. 6:**
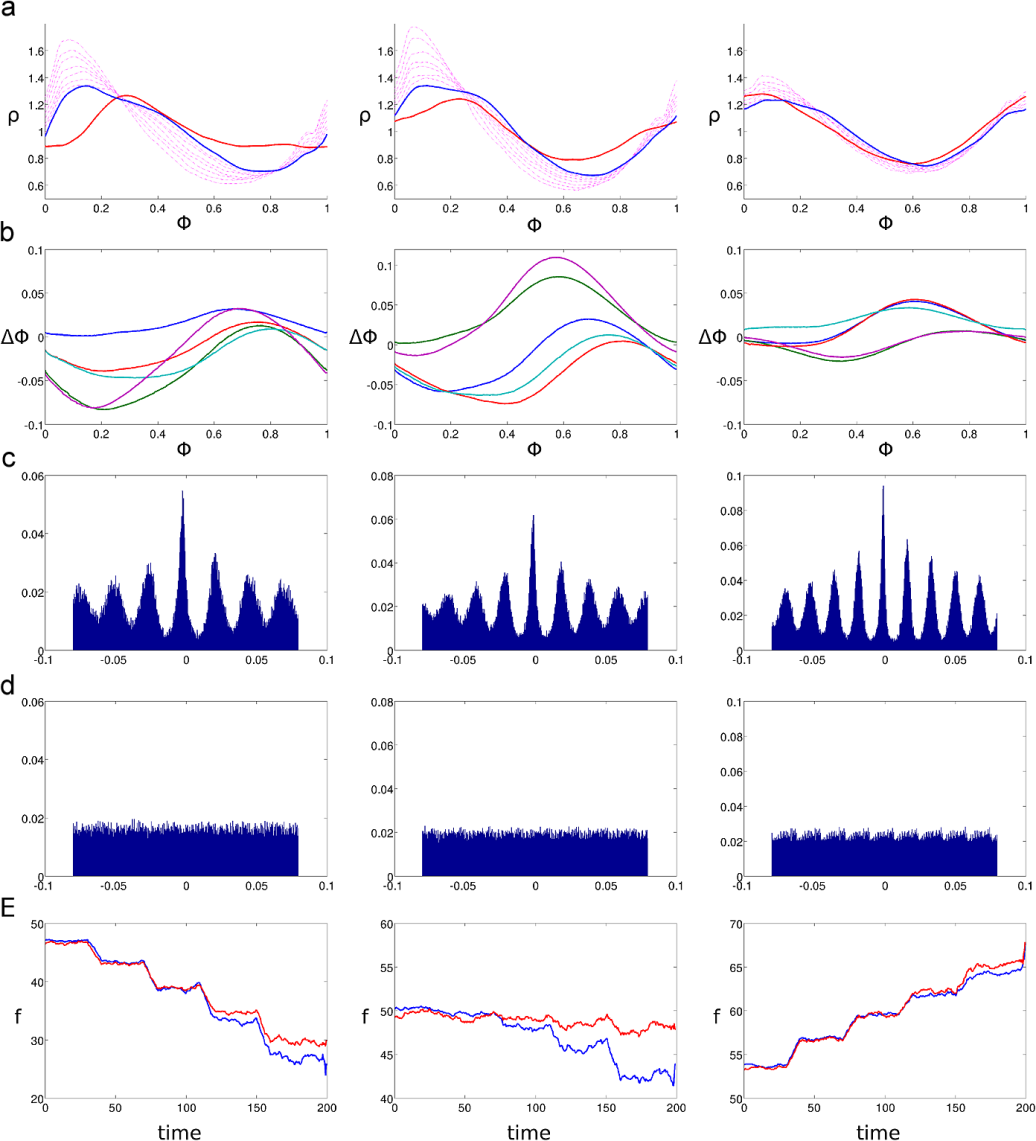
Performance of the fifth equation, PRC version (heterogeneous inputs) with 5 ms delay. Simulations for three different combinations of mean PRC amplitudes. For each simulation a total of $N_{\mathrm{syn}}=60$ different inputs were used, each with randomly chosen amplitudes $a_{\mathrm{exc}}^{j}$ and $a_{\mathrm{inh}}^{j}$ for $\varDelta _{\mathrm{exc}}^{j}$ and $\varDelta _{\mathrm{inh}}^{j}$. The firing rate for all inputs was $r_{j} = 10\mbox{ Hz}$. *The figures on the left* correspond to a simulation where the mean of the PRC amplitudes is $\langle a_{\mathrm{exc}}^{j}\rangle = 1/30$, $\langle a_{\mathrm{inh}}^{j}\rangle = 2/30$, *the figures in the middle* correspond to $\langle a_{\mathrm{exc}}^{j}\rangle = 2/30$, $\langle a_{\mathrm{inh}}^{j}\rangle = 2/30$, and *the figures on the right* correspond to $\langle a_{\mathrm{exc}}^{j}\rangle = 2/30$, $\langle a_{\mathrm{inh}}^{j}\rangle = 1/30$. **a**: Stationary phase PDFs for the oscillator with feedforward inhibition (*red*), and with the fifth version of the equivalent PRCs (*blue*). *Magenta dash-dotted lines* indicate the phase PDF corresponding to intermediate iterations in the iterative procedure used. **b**: PRCs used in the simulation. *Different colored lines* show the equivalent PRCs for the first five synapses. **c**: Cross-correlogram between the output spike trains of the oscillators with feedforward inhibition and with the equivalent PRCs. *The horizontal axis* indicates time shift, and *the vertical axis* the normalized spike count. Normalization was done by dividing the bin spike counts between the total number of spikes in the output of the oscillator with two PRCs. **d**: Cross-correlograms between the output spike train of the oscillator with feedforward inhibition and a regular spiking oscillator matching its mean frequency. Normalization of the spike counts per bin was done as in **c**. **e**: Firing rate response of the oscillators with feedforward inhibition (*red*), and the equivalent PRCs (*blue*) to five different levels of input rates. Input rates range from 240 Hz to 1200 Hz in constant increments of 240 Hz

The next result shown is for the second and third versions of $\varDelta _{\mathrm{eq}}$. The third version of the equivalent PRC can give accurate results in replicating the response of an oscillator with feedforward inhibition, especially when the feedforward delay is small. If this delay is small enough, even the second version of the equivalent PRC (Eq. (23)) can give reasonable results. This is seen in Fig. 4, where the feedforward delay is taken to be 1 ms. The simulations used to produce Figs. 4, 5, and 6 were similar to those presented in the previous subsection, the only difference is in the equivalent PRCs used, and in the case of Fig. 4, the length of the feedforward delay.

In panel a of Figs. 4, 5, and 6, it is again seen that the matching of stationary phase PDFs is not as good as in the previous case (shown in Fig. 2), when the formulas where derived for this purpose. This is to be expected; for each excitatory input the oscillator with feedforward inhibition advances its phase, whereas the oscillator with the second and third equivalent PRCs experience a shift in phase that includes an estimate of future inhibition. On the other hand, when tracking the timing of output spikes, the oscillator with the third version of the PRC tends to outperform the oscillator with a phase PDF-matching PRC. In panel d of Fig. 4, the normalized cross-correlogram shows a large similarity between the output spike trains. Simulations with the first equivalent PRC do not produce such a good cross-correlogram, even when the feedforward delay is 1 ms (data not shown). As before, to show that the peaks in the cross-correlogram come from a similar temporal structure in the spikes from both oscillators, and not just from them having similar periods, I also show in panel e the cross-correlogram between the spike train with delayed inhibitory inputs, and a spike train with constant interspike intervals and the same frequency.

Another relevant result is in panel c of Fig. 4. This panel shows a close agreement between the expected inhibitory shift at time $t+d$ obtained from simulations (red curve), and from Eq. (27) (black curve), which approximates the expected inhibition by assuming that during the inhibitory delay all inputs will arrive at regular intervals.

Reported values of delay between an excitatory input and the corresponding feedforward inhibition are usually in the 1–2 ms range [36, 59], and have even been described as non-existent [61]. It is nevertheless relevant to test whether the formulas of this paper can be still applicable when the feedforward delay is not as short. The approaches taken here to derive the equivalent PRCs should show their shortcomings as the delay and the input firing rate are increased. With this in mind the simulations in this paper—with the exception of the one in Fig. 4—use a delay of 5 ms, larger than reported values, but still biologically plausible.

Figure 5 illustrates simulations done with the second and third versions of $\varDelta _{\mathrm{eq}}$ and a feedforward delay of 5 ms. It is evident in panel e that the second version of the PRC becomes incapable of matching the firing rate of the oscillator with feedforward inhibition as the input rates become larger. On the other hand, the oscillator with the third version of $\varDelta _{\mathrm{eq}}$ still displays similar behavior to the oscillator with feedforward inhibition. It should be noted that the third PRC version only requires to specify three parameters: the maximum number of inputs to be considered during the delay period (limiting the number of terms in Eq. (24)), and for the iterative procedure, the number of iterations *M*, and the learning rate *ϵ*. In the case shown in Fig. 5, there were up to seven inputs during the delay period, and $M=10$, $\epsilon=0.04$. For the case shown in Fig. 3, up to eight inputs were considered during the delay period, with $M=18$, $\epsilon=0.02$.

The fifth version of the equivalent PRC was also tested, using 60 different inputs, each one with different amplitudes for its excitatory and inhibitory PRCs. The result of the simulations is illustrated in Fig. 6. As can be seen from this figure, and from Fig. 5, the performance for oscillators with homogeneous and heterogeneous inputs is very similar. The fifth PRC version uses the same three parameters as the third version, and optionally, a batch size for the iterative procedure. For the simulation in Fig. 6, $M=10$, $\epsilon=0.04$, and up to three inputs were considered during the delay period.

## Discussion

Experimental results point to the possibility of Purkinje cells synchronizing due to correlated inputs. Comodulation of firing rates is insufficient to explain this, but we can resort to the emerging mathematical theory of stochastic synchronization. At the outset, however, it is unclear that this theory is applicable here due to the presence of feedforward inhibition. Rather than obtaining a theory of how uncoupled oscillators with feedforward inhibition synchronize, I suggest to define equivalent PRCs meant to show that the systems with feedforward inhibition, which have excitatory and inhibitory PRCs, behave in a similar way to a system with only one PRC. The equivalent PRC is what in practice can be used to study the synchronization properties of oscillators. The first type of equivalent PRC defined here can be used to produce any given phase PDF (assuming Poisson inputs), and in particular, can produce the phase PDF that arises from a system with feedforward inhibition. With the second type of PRC defined here we can obtain an oscillator that, on average, has the same phase as the oscillator with feedforward inhibition after receiving the inhibitory stimulus. Unlike the first type of equivalent PRC, the second one requires knowing the shape of the excitatory and inhibitory PRCs in order to be computed. The phase PDF, excitatory PRC, and inhibitory PRC of Purkinje cells can all be experimentally measured, although the exact methodology is beyond the scope of this paper.

An interesting insight arising from the equivalent PRC is that feedforward inhibition could have the effect of enhancing synchronization. Type II PRCs, which are positive for late phases and negative for early phases [57], are the best at synchronizing due to correlated inputs [41, 42, 44, 46, 48]. As can be observed from Figs. 3, 4, 5, 6, feedforward inhibition has the effect of adding a negative region to the equivalent PRC which makes it of type II. Although the shape of inhibitory PRCs is still unknown, it seems reasonable that this result will hold for most reasonable shapes, inhibition delays, and ratios of excitatory to inhibitory amplitudes.

A final observation is that comparing the results from the two types of equivalent PRCs (Fig. 3) leads to the curious conjecture that an optimal equivalent PRC (in the sense of maximizing coherence with the output spike train of the oscillator with feedforward inhibition) need not also produce a matching phase PDF.

### Is the PRC Model Adequate?

Purkinje cells are particularly complex, and their computational models tend to be mathematically intractable. The oscillator representation is a mathematically tractable model that allows one to construct complicated hypotheses that may then be addressed by physiological experiments and by detailed models. This extra step in the modeling process is beneficial in the study of synchronization because the effects of changing physiological parameters are not straightforward in this case. Still, modeling Purkinje cells as oscillators and characterizing their inputs through a PRC entails a drastic reduction in complexity, which could obscure important details. Purkinje cells tonically fire simple spikes with frequencies ranging from 30 to 150 Hz; these frequencies are modulated by afferent and efferent inputs (e.g. [62, 63]), creating a wide dynamic range. It is likely, however, that Purkinje cells are intrinsically regular, and if they show irregular inter-spikes intervals in vivo, this is because of MLI inputs ([37]). Moreover, we may not need to consider the complex morphology of PC dendritic trees, as the summation of inputs may happen independently of their location and distribution ([64]). Indeed, in the dendritic tree there exist voltage-gated calcium channels that amplify distant focal inputs more than proximal ones, canceling cable attenuation and making the somatic response largely independent of input location.

Further considerations regarding the general suitability of the PRC representation are presented in [54]. For the specific case of Purkinje cells, the main concerns when using the theory of stochastic synchronization may be the variability in firing rates, and the fact that the PRCs change depending on this firing rate. These issues are not fully resolved, and a task for the future may be to test possible answers to them.

It is the author’s opinion that the results in this paper will become relevant only if synchronization by virtue of correlated inputs is found to exist to a significant degree among Purkinje cells projecting to the same nuclear cell. This may be the next question to be addressed, and it can start by working with more realistic computational models of Purkinje cells. Realistic modeling of cerebellar cells has been taking place for decades, and there are several models that could be used for this endeavor (e.g. [25, 38, 65, 66]). These models can point out possible necessary conditions for the granule layer inputs, Purkinje cells, and molecular layer interneurons in order to express stochastic synchronization. These conditions can then be explored experimentally.
